# Supplementary material for: Nesting of groups: Links among personal magnetism, community trust, inequality perception and depression
Source: PLoS One. 2025 Mar 25;20(3):e0320560. doi: 10.1371/journal.pone.0320560 (PMC11936175; doi:10.1371/journal.pone.0320560)
Supplement: Attachment — Submitted filename: PONE-D-24-32639_Reviewed_article.pdf [file pone.0320560.s001.pdf]

PLOS ONE

Nesting of groups: Links among personal magnetism, community trust, inequality perception and depression

--Manuscript Draft--

**Manuscript Number:**	PONE-D-24-32639
**Article Type:**	Research Article
**Full Title:**	Nesting of groups: Links among personal magnetism, community trust, inequality perception and depression
**Short Title:**	Nesting of groups
**Corresponding Author:**	Yujie Zhang<br>Chongqing University<br>Chongqing, CHINA
**Keywords:**	personal magnetism; depression mitigation; income inequality perception; community trust dynamics; societal transition in China
**Abstract:**	Background<br>Amidst China's social transition from a collectivist 'Danwei' system to a community-oriented society, this study probes the nexus between personal magnetism—a constellation of an individual's social allure—and its mitigating influence on depression.<br>Objective<br>The study scrutinizes the moderating role of inequality perception and the mediating effects of community trust within this dynamic.<br>Methods<br>Analyzing longitudinal data from the China Family Panel Studies spanning 2018 to 2020, encompassing 26,711 individuals aged 16 to 96, the study deploys a multivariate regression model for empirical tests.<br>Results<br>Findings elucidate that inequality perception not only strengthens the inverse relationship between personal magnetism and depression but also highlights community trust as a pivotal mediating variable. More importantly, this study presents a nuanced examination of how perceived income inequality modulates the inverse relationship between personal magnetism and depression. It substantiates the principles of inequity aversion theory, proposing that an acute consciousness of economic disparities catalyzes a shift toward prosocial activities. Such behaviors are conducive to the alleviation of depressive symptoms, as they foster community cohesion and individual well-being.<br>Conclusion<br>These findings advocate for the enhancement of social policies that foster community solidarity and address income inequality perceptions, thereby leveraging the protective properties of personal magnetism to alleviate depression.
**Order of Authors:**	Peicheng Wang
	Yujie Zhang, Ph.D.
**Additional Information:**	
**Question**	**Response**
**Financial Disclosure**<br><br>Enter a financial disclosure statement that describes the sources of funding for the work included in this submission. Review the submission guidelines for detailed requirements. View published research articles from *PLOS ONE* for specific examples.	Yes

Powered by Editorial Manager® and ProduXion Manager® from Aries Systems Corporation

This statement is required for submission and **will appear in the published article** if the submission is accepted. Please make sure it is accurate.<br><br>**Funded studies**<br>Enter a statement with the following details:<br>• Initials of the authors who received each award<br>• Grant numbers awarded to each author<br>• The full name of each funder<br>• URL of each funder website<br>• Did the sponsors or funders play any role in the study design, data collection and analysis, decision to publish, or preparation of the manuscript?<br><br>Did you receive funding for this work?	
Please add funding details.<br>as follow-up to "**Financial Disclosure**<br><br>Enter a financial disclosure statement that describes the sources of funding for the work included in this submission. Review the submission guidelines for detailed requirements. View published research articles from *PLOS ONE* for specific examples.<br><br>This statement is required for submission and **will appear in the published article** if the submission is accepted. Please make sure it is accurate.<br><br>**Funded studies**<br>Enter a statement with the following details:<br>• Initials of the authors who received each award<br>• Grant numbers awarded to each author<br>• The full name of each funder<br>• URL of each funder website<br>• Did the sponsors or funders play any role in the study design, data collection and analysis, decision to publish, or preparation of the manuscript?	This study was supported by Chongqing Municipal Social Science Planning Doctoral and Cultivation Project [Grant 2023BS080].

Did you receive funding for this work?"	
Please select the country of your main research funder (please select carefully as in some cases this is used in fee calculation).<br>as follow-up to "**Financial Disclosure**<br><br>Enter a financial disclosure statement that describes the sources of funding for the work included in this submission. Review the submission guidelines for detailed requirements. View published research articles from *PLOS ONE* for specific examples.<br><br>This statement is required for submission and **will appear in the published article** if the submission is accepted. Please make sure it is accurate.<br><br>**Funded studies**<br>Enter a statement with the following details:<br>• Initials of the authors who received each award<br>• Grant numbers awarded to each author<br>• The full name of each funder<br>• URL of each funder website<br>• Did the sponsors or funders play any role in the study design, data collection and analysis, decision to publish, or preparation of the manuscript?<br><br>Did you receive funding for this work?"	CHINA - CN
**Competing Interests**<br><br>Use the instructions below to enter a competing interest statement for this submission. On behalf of all authors, disclose any competing interests that could be perceived to bias this work—acknowledging all financial support and any other relevant financial or non-financial competing interests.<br><br>This statement is required for submission	The authors have declared that no competing interests exist.

and **will appear in the published article** if the submission is accepted. Please make sure it is accurate and that any funding sources listed in your Funding Information later in the submission form are also declared in your Financial Disclosure statement.<br><br>View published research articles from *PLOS ONE* for specific examples.<br><br>**NO authors have competing interests**<br><br>Enter: *The authors have declared that no competing interests exist.*<br><br>**Authors with competing interests**<br><br>Enter competing interest details beginning with this statement:<br><br>*I have read the journal's policy and the authors of this manuscript have the following competing interests: [insert competing interests here]*<br><br>* typeset	
**Ethics Statement**<br><br>Enter an ethics statement for this submission. This statement is required if the study involved:<br><br>• Human participants<br>• Human specimens or tissue<br>• Vertebrate animals or cephalopods<br>• Vertebrate embryos or tissues<br>• Field research<br><br>Write "N/A" if the submission does not require an ethics statement.<br><br>General guidance is provided below. Consult the submission guidelines for detailed instructions. **Make sure that all information entered here is included in the Methods section of the manuscript.**	N/A

**Format for specific study types**<br><br>**Human Subject Research (involving human participants and/or tissue)**<br>• Give the name of the institutional review board or ethics committee that approved the study<br>• Include the approval number and/or a statement indicating approval of this research<br>• Indicate the form of consent obtained (written/oral) or the reason that consent was not obtained (e.g. the data were analyzed anonymously)<br><br>**Animal Research (involving vertebrate animals, embryos or tissues)**<br>• Provide the name of the Institutional Animal Care and Use Committee (IACUC) or other relevant ethics board that reviewed the study protocol, and indicate whether they approved this research or granted a formal waiver of ethical approval<br>• Include an approval number if one was obtained<br>• If the study involved *non-human primates*, add *additional details* about animal welfare and steps taken to ameliorate suffering<br>• If anesthesia, euthanasia, or any kind of animal sacrifice is part of the study, include briefly which substances and/or methods were applied<br><br>**Field Research**<br><br>Include the following details if this study involves the collection of plant, animal, or other materials from a natural setting:<br>• Field permit number<br>• Name of the institution or relevant body that granted permission	
**Data Availability**<br><br>Authors are required to make all data underlying the findings described fully available, without restriction, and from the time of publication. PLOS allows rare exceptions to address legal and ethical concerns. See the PLOS Data Policy and FAQ for detailed information.	Yes - all data are fully available without restriction

A Data Availability Statement describing where the data can be found is required at submission. Your answers to this question constitute the Data Availability Statement and **will be published in the article**, if accepted.<br><br>**Important:** Stating 'data available on request from the author' is not sufficient. If your data are only available upon request, select 'No' for the first question and explain your exceptional situation in the text box.<br><br>Do the authors confirm that all data underlying the findings described in their manuscript are fully available without restriction?	
**Describe where the data may be found in full sentences. If you are copying our sample text, replace any instances of XXX with the appropriate details.**<br><br>• If the data are **held or will be held in a public repository**, include URLs, accession numbers or DOIs. If this information will only be available after acceptance, indicate this by ticking the box below. For example: *All XXX files are available from the XXX database (accession number(s) XXX, XXX.).*<br>• If the data are all contained **within the manuscript and/or Supporting Information files**, enter the following: *All relevant data are within the manuscript and its Supporting Information files.*<br>• If neither of these applies but you are able to provide **details of access elsewhere**, with or without limitations, please do so. For example:<br><br>*Data cannot be shared publicly because of [XXX]. Data are available from the XXX Institutional Data Access / Ethics Committee (contact via XXX) for researchers who meet the criteria for access to confidential data.*<br><br>*The data underlying the results presented in the study are available from (include the name of the third party*	The data of CFPS is publicly available at https://doi.org/10.18170/DVN/45LCSO

and contact information or URL).<br>• This text is appropriate if the data are owned by a third party and authors do not have permission to share the data.<br><br>* typeset	
Additional data availability information:	Tick here if the URLs/accession numbers/DOIs will be available only after acceptance of the manuscript for publication so that we can ensure their inclusion before publication.

Powered by Editorial Manager® and ProduXion Manager® from Aries Systems Corporation

Manuscript

Click here to access/download;Manuscript;0804Revised Manuscript.docx

Nesting of groups: Links among personal magnetism, community trust, inequality perception and depression

Peicheng Wang[1], Yujie Zhang[2,*]

[1]School of International and Public Affairs, Shanghai Jiao Tong University, No. 1954 Huashan Road, Xuhui District, Shanghai 200030, China; E-mail: wangpeicheng@sjtu.edu.cn

[2]School of Public Policy and Administration, Chongqing University, No. 174 Shazheng Street, Shapingba District, Chongqing 400044, China; E-mail: zhangyujie33@foxmail.com/zhangyujie@cqu.edu.cn

***Corresponding author: Yujie Zhang**

Complete address: School of Public Policy and Administration, Chongqing University, Shazheng Street No.174, Shapingba District, Chongqing, China.

Institutional e-mail address: zhangyujie@cqu.edu.cn

Personal e-mail address: zhangyujie33@foxmail.com

Ethical approval

All procedures performed in the study involving human participants were in accordance with the ethical standards of the institutional and/or national research committee and with the 1964 Helsinki declaration and its later amendments or comparable ethical standards. The survey was also conducted within the article 38, 39, and 40 of the Constitution of People's Republic of China and the legal framework governed by Chapter I, Article 9 from the statistics law of the People's Republic of China. Research ethics approval was received from the Peking University (Ethical Number:

IRB00001052-14010).

Informed consent

Verbal informed consent was obtained from all individual participants included in the study. The design of this survey was within the article 38, 39, and 40 of the Constitution of People's Republic of China and the legal framework governed by Chapter I, Article 9 from the statistics law of the People's Republic of China. Verbal informed consent was acceptable and was not reviewed by ethics committee. Moreover, the interviewer also had documented the more details information on the process of obtaining informed consent, which included whether agree to attend this study, the time of agree to attend this study, the reasons of disagree to attend this study, and so on. Details of informed consent was stored by the Peking University.

Consent for publication

Not applicable.

Data availability

The data of CFPS is publicly available at https://doi.org/10.18170/DVN/45LCSO accessed on 25 April 2023.

Funding

This study was supported by Chongqing Municipal Social Science Planning Doctoral and Cultivation Project [Grant 2023BS080].

Competing interests

The authors declared no potential conflicts of interest with respect to the research, authorship, and/or publication of this article.

Author contributions

The authors collaboratively and equally contributed to the study conceptualization, data acquisition, methodology development, data analysis, draft writing, and data interpretation.

Acknowledgment

The author would like to acknowledge the CFPS team for providing the data.

Biographical note

Peicheng Wang is a doctoral candidate at the School of International and Public Affairs at Shanghai Jiao Tong University. His research domain lies in mental health studies, with a particular emphasis on depression.

Yujie Zhang is an assistant professor at the School of Public Policy and Administration at Chongqing University. She earned her Ph.D. in Public Administration from Shanghai Jiao Tong University. Her primary research areas are civic participation and political psychology. Her recent publications in International Sociology and Anthropological Forum focus on cooperative governance and social mentality.

Abstract

Background

Amidst China's social transition from a collectivist 'Danwei' system to a community-oriented society, this study probes the nexus between personal magnetism—a constellation of an individual's social allure—and its mitigating influence on depression.

Objective

The study scrutinizes the moderating role of inequality perception and the mediating effects of community trust within this dynamic.

Methods

Analyzing longitudinal data from the China Family Panel Studies spanning 2018 to 2020, encompassing 26,711 individuals aged 16 to 96, the study deploys a multivariate regression model for empirical tests.

Results

Findings elucidate that inequality perception not only strengthens the inverse relationship between personal magnetism and depression but also highlights community trust as a pivotal mediating variable. More importantly, this study presents a nuanced examination of how perceived income inequality modulates the inverse relationship between personal magnetism and depression. It substantiates the principles of inequity aversion theory, proposing that an acute consciousness of economic disparities catalyzes a shift toward prosocial activities. Such behaviors are conducive to the alleviation of depressive symptoms, as they foster community cohesion and individual well-being.

Conclusion

These findings advocate for the enhancement of social policies that foster community solidarity and address income inequality perceptions, thereby leveraging the protective properties of personal magnetism to alleviate depression.

Keywords

personal magnetism; depression mitigation; income inequality perception; community

trust dynamics; societal transition in China

Introduction

As China acceded to the World Trade Organization, it embarked on an unprecedented trajectory of economic expansion that has not only reshaped its urban landscape but also instigated a profound recalibration of its social fabric [1]. The urban metamorphosis has sown seeds of socioeconomic disparity, fraying the tightly woven communal networks that once anchored Chinese neighborhoods [2]. This societal upheaval has amplified concerns surrounding depression, a burgeoning public health crisis, as evidenced by the 6.8% lifetime prevalence of depressive disorders among adults, and the concomitant low treatment engagement within the nation [3].

Literature review

Personal magnetism and depression

The construct of personal magnetism occupies a pivotal role in the psychological discourse, particularly as it pertains to its potential as a buffer against depressive symptoms. Rooted in the principles of social psychology, personal magnetism is conceptualized as an individual's social allure and influence—a quality that inherently fosters robust interpersonal connections. These connections are not merely superficial ties; they imbue individuals with a sense of worth and belonging, functioning as critical psychological resources in the face of adversity [4, 5].

Research has illuminated the protective effects of personal magnetism against depressive symptoms, underscoring the importance of social bonds in maintaining mental health. For instance, a study by Kawachi and Berkman posits that the breadth

and depth of one's social networks—elements central to the concept of personal magnetism—can mitigate the impact of stressors that often precipitate depression [6]. Similarly, Cacioppo and Hawkley have identified the experience of loneliness, essentially the antithesis of personal magnetism, as a significant risk factor for depression, further highlighting the importance of social connectedness [7].

In transitioning societies like China, where rapid socio-economic changes have disrupted traditional social networks, personal magnetism gains even greater significance. The dissolution of the 'Danwei' system, characterized by integrated work units that provided social stability, has left a void that personal magnetism is well-positioned to fill [8, 9]. This societal transformation has necessitated new means of social integration, with personal magnetism emerging as a valuable asset in the formation of new social ties and the preservation of mental health [10].

Predicated on the idea that individuals with higher personal magnetism are able to forge and sustain meaningful social connections, which serve as a bulwark against the psychological distress that can lead to depression [11, 12], this study proposes:

Hypothesis 1: Personal magnetism is negatively correlated with depression.

The role of perceived inequality

Building upon the theoretical framework that personal magnetism serves as a bulwark against depression, this study probes deeper into the societal undercurrents that may intensify this dynamic. The hypothesis posits that an individual's perception of income inequality can act as a catalyst, amplifying the protective attributes of personal magnetism. This concept aligns with the theory of inequity aversion, where higher

awareness of societal disparities drives individuals toward compensatory prosocial behaviors [13, 14]. These behaviors, rooted in the fundamental human desire for fairness and equity, could enhance interpersonal relationships and, subsequently, an individual's social net worth or personal magnetism [15, 16]. As such, an individual's perception of their social environment's fairness may be as critical to their mental health as their immediate social interactions.

Furthermore, the study leverages the concept of relative deprivation—a psychological state resulting from the perception of unfair economic disparity—which can lead to feelings of discontent and resentment [17, 18]. It is posited that personal magnetism, which can be understood as a form of social capital, may mitigate the negative psychological impact of perceived relative deprivation. Individuals who feel socially valued and connected are likely to have a buffer against the adverse effects of perceiving themselves as economically disadvantaged [19, 20]. In essence, the study suggests that the social fabric's integrity, as perceived by individuals, plays a pivotal role in mental health outcomes, moderating the effects of personal social resources.

The interplay of personal magnetism and perceived income inequality extends to the domain of social comparison, where individuals evaluate their own socio-economic standing in relation to others. According to Festinger's social comparison theory [21], people have an inherent drive to evaluate themselves, often in comparison with others. In a society where income disparities are pronounced, those with strong personal magnetism may utilize their social clout to navigate these disparities more effectively, leading to better mental health outcomes [22]. As such, personal magnetism could

compensate for the psychological discomfort engendered by unfavorable social comparisons, reducing the likelihood of depression among those with higher social standing. This complex interrelation between individual psychological processes and socio-economic structures forms the crux of this study's second hypothesis, presenting a multifaceted view of the determinants of mental health in contemporary China [23, 24]. Hence, this study proposes:

Hypothesis 2: Inequality perception strengthens the relationship between personal magnetism and depression.

The role of community trust

The intricate nexus between personal magnetism, community trust, and depression unveils the dynamic interplay of individual characteristics and social structures within the context of mental health. This study delves into the mediating role of community trust in the relationship between personal magnetism and depression, positing that individuals with higher levels of personal magnetism are likely to be perceived as more trustworthy, fostering stronger community bonds and facilitating social cohesion [25]. This perspective is buttressed by the social capital theory, which suggests that trust—both interpersonal and communal—serves as a fundamental component of social capital, crucial for psychological well-being and the prevention of mental health disorders [26].

Moreover, the transformation of Chinese society from a 'Danwei'-based system to a community-centric structure underscores the growing importance of personal magnetism in establishing trust within communities increasingly characterized by anonymity [27]. As individuals navigate this new societal landscape, the emergence of

trust networks is hypothesized to mediate the impact of personal magnetism on depression, suggesting that the formation of trust can significantly dampen the adverse effects of low personal magnetism on mental health [6, 19]. This mediation is likely a reflection of the protective role that social connections and perceived social support play in the mental health of individuals, as posited in the buffer theory [28, 29].

In synthesizing these insights, this study advances a theoretical framework that interlaces personal magnetism with community trust and depression, illuminating a potential mechanism through which personal magnetism exerts its influence. This framework aligns with the broader literature on social determinants of health, which recognizes the centrality of social relationships and community engagement in shaping health outcomes [30, 31]. By integrating community trust as a mediating variable, the study enriches the discourse on the psychosocial pathways that mitigate depression, positing a refined model wherein personal magnetism enhances community trust, which, in turn, mitigates depression—a pathway that holds profound implications for social work in public health and the development of community-based mental health interventions [32]. Hence, this study proposes:

Hypothesis 3: Community trust mediates the relationship between personal magnetism and depression.

Thus, this study contributes to a nuanced understanding of the mechanisms at play within China's social transformation. It underscores the importance of examining not only the direct effects of personal magnetism on depression but also the intricate interplay of societal perceptions and community dynamics that modulate this

relationship. By exploring these undercurrents, the study aims to illuminate potential pathways for policy interventions that nurture community trust and address income disparities, thereby leveraging personal magnetism as a bulwark against depression in China's new societal paradigm. Figure 1 presents the study's research framework.

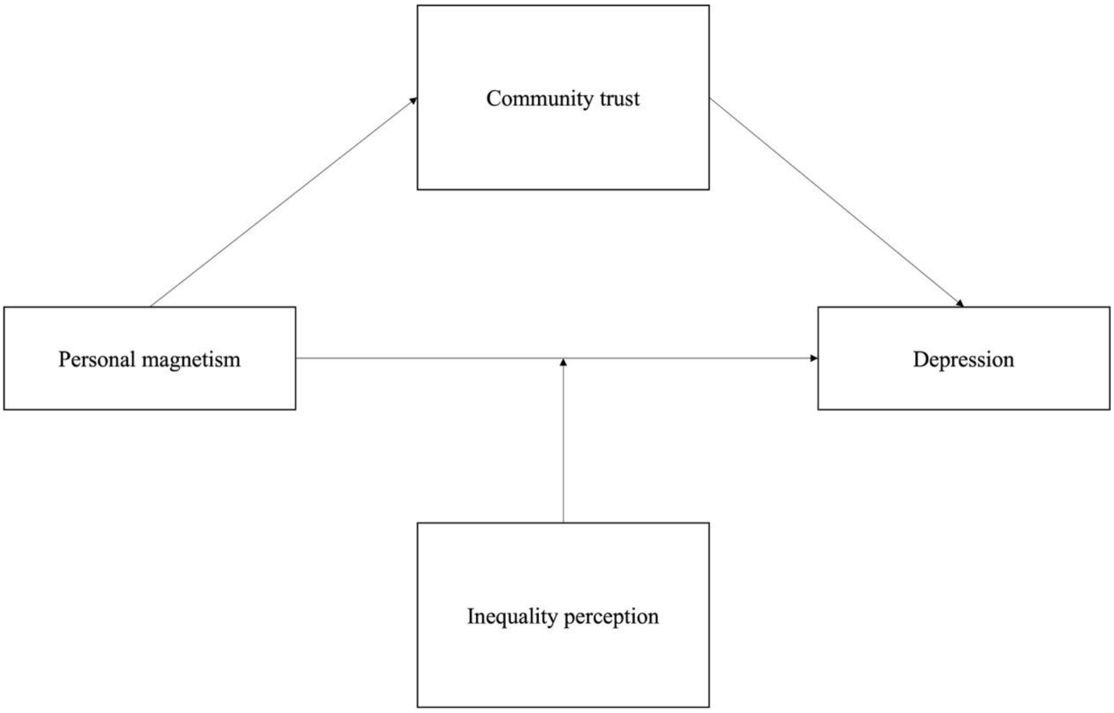

Figure 1. The research framework of the relationship between personal magnetism and depression.

Methods

Data and sample

Utilizing data from the 2018 and 2020 waves of the China Family Panel Studies (CFPS), this study capitalizes on a longitudinal and nationally representative sample to investigate the interplay between personal magnetism, perceived income inequality, community trust, and depression. The CFPS, helmed by the Institute of Social Science Survey at Peking University, employs a rigorous stratified random sampling approach, capturing a broad demographic swath across 25 provinces, municipalities, and

autonomous regions. The CFPS's extensive reach into 16,000 households provides a rich tapestry of the Chinese societal landscape. The meticulous data gathering executed by trained interviewers using computer-assisted personal interviewing ensures the robustness and precision of the dataset. After careful data curation to exclude incomplete records, the analytical sample comprised 26,714 respondents, offering a substantial base for comprehensive societal analysis.

Research ethics approval was received from the Peking University Ethics Committee. Verbal informed consent was obtained from all individual participants included in the study. The process for obtaining verbal consent was as follows: participants were provided with a detailed explanation of the study's purpose, procedures, potential risks, and benefits. They were then asked to verbally confirm their willingness to participate. This verbal consent process was documented by recording the consent conversations with participants, which were then transcribed and securely stored. A witness was present during each consent conversation to ensure the accuracy and validity of the documentation.

Measures

Depression

In the CFPS, depression was gauged using an adaptation of the Center for Epidemiologic Studies Depression Scale (CES-D), a tool previously validated for its high reliability and validity within the Chinese demographic [33,34]. This adapted version solicited responses from participants via a series of six questions designed to evaluate depression: frequency of feeling depressed, difficulty in initiating activities,

sleep disturbances, experiencing unpleasant moods, feelings of loneliness, and pervasive unhappiness. The questions were measured on a Likert scale ranging from 1 (indicating the rarity of such feelings) to 4 (indicating such feelings occur almost every day). To maintain objectivity and preclude subjective biases in classifying depression, the aggregate score of these questions was employed as a quantitative representation of depression severity. The reliability of this depression scale was confirmed within the study, with a Cronbach's alpha coefficient of 0.76, indicating a commendable level of internal consistency.

Personal magnetism

Within the framework of the CFPS, the construct of personal magnetism was quantified through a single-item Likert-scale metric [35, 36]. Participants were instructed to self-assess their level of personal magnetism on a scale where a rating of point 0 corresponded to the least popular and a rating of point 10 signified the most popular. This scalar approach to measuring personal magnetism provides a straightforward yet effective means of capturing self-perceived social standing within the community.

Inequality perception

The assessment of inequality perception within the context of the CFPS was operationalized using a Likert-scale instrument [37]. Participants were requested to evaluate their perception of income disparity, with the scale extending from point 0, denoting a perception of very low inequality, to point 10, indicating a perception of very high inequality. This metric facilitates the quantification of individuals' subjective experiences of income distribution within their social milieu.

Community trust

The construct of community trust was quantified in the CFPS utilizing a Likert-scale measure [38]. Participants were prompted to appraise the level of trust within their community, with the scale ranging from point 0, signifying very low trust, to point 10, signifying very high trust. This instrument serves to measure the subjective trustworthiness perceived by individuals within their community networks.

Control variables

The operationalization of control variables in this study was informed by extant scholarly works [39, 40]. These variables encompass sex, coded as 0 for female and 1 for male; age, treated as a continuous variable; marital status, with 0 denoting unmarried and 1 denoting married; educational attainment, categorized into three levels—1 for education below junior high school, 2 for education spanning junior high school to college, and 3 for education beyond college level; self-assessed health status, scaled from 1 representing 'unhealthy' to 5 representing 'very healthy'; smoking habits, coded as 0 for non-smokers and 1 for smokers; alcohol consumption, coded as 0 for non-drinkers and 1 for drinkers; and type of household registration, with 0 indicating rural and 1 indicating urban registration. The descriptive statistics for all variables are presented in Table 1.

Table 1. Descriptive statistics of all variables, CFPS 2018-2020 (N = 26,711).

**Variable**	**Number of observations (Proportion) / Mean (SD)**
Dependent variable	

Depression	13.433 (3.720)
Independent variable	
Personal magnetism	6.987 (1.796)
Moderating variable	
Inequality perception	7.189 (2.269)
Mediating variable	
Community trust	6.556 (2.035)
Control variables	
Sex (%)	
Female	13,409 (50.20%)
Male	13,302 (49.80%)
Age	33.525 (11.889)
Marital status (%)	
Unmarried	8,553 (32.02%)
Married	18,158 (67.98%)
Education (%)	
Below junior high school	6,242 (23.37%)
Between junior high school and college	17,487 (65.47%)
Above college	2,982 (11.16%)
Self-rated health	3.326 (1.095)
Smoking (%)	

Non-smoker	19,480 (72.93%)
Smoker	7,231 (27.07%)
Alcohol drinking (%)	
Non-drinker	23,678 (88.65%)
Drinker	3,033 (11.35%)
Residence registration (%)	
Rural	19,957 (74.71%)
Urban	6,754 (25.29%)

Statistical analysis

This study employed Stata version 16 (StataCorp, Texas, USA) for its data analysis procedures. First, this study conducted a multicollinearity diagnostic using variance inflation factors (VIF), with all VIFs registering below 2, thereby indicating an absence of significant multicollinearity within the variables. Second, multivariate regression modeling techniques were subsequently utilized to explore potential moderating and mediating influences [41]. To mitigate multicollinearity concerns, the independent variables and moderators were mean-centered prior to analysis [42]. The Bootstrap method was harnessed to ascertain the presence and extent of mediation effects [43]. Third, this study undertook a heterogeneity examination contingent upon sex. Robustness checks were meticulously carried out to ensure the validity of the findings.

Results

Multivariate regression analysis

Table 1 reveals insights into the state of mental health and its sociodemographic

correlations. The mean depression score across the sample stands at 13.433 (S. D. = 3.720), highlighting a moderate level of depressive symptoms. This is juxtaposed with the mean personal magnetism score of 6.987 (S. D. = 1.796), suggesting a relatively high perceived social allure among participants. The data on inequality perception reveal a mean of 7.189 (S. D. = 2.269), indicating noticeable awareness of socioeconomic disparities, while community trust averages at 6.556 (S. D. = 2.035), reflecting a moderate level of trust within communities.

The control variables offer a demographic breakdown: females make up 50.20% of the sample, indicating a near-equitable sex distribution. The average age is 33.525 years (S. D. = 11.889), encompassing a wide age range among participants. Marital status shows that a majority, 67.98%, are married, and educational levels vary, with 65.47% falling between junior high school and college education. The sample's health perception is relatively positive, with a mean self-rated health score of 3.326 (S. D. = 1.095). Smoking and alcohol drinking habits are less prevalent, with 27.07% identifying as smokers and 11.35% as drinkers. The majority of participants are from rural areas (74.71%), which may influence the interpretation of community trust and mental health outcomes, given the distinct social structures in rural settings. These descriptive findings provide a solid foundation for understanding the context of the studied mental health variables within the broader Chinese sociodemographic landscape.

Table 2 provides a nuanced picture of how inequality perception interacts with personal magnetism to influence depression. The multivariate regression analysis in

Step 1 reveals a significant negative effect of personal magnetism on depression (Coef. = -0.113, $p < 0.001$), suggesting that individuals with higher personal magnetism experience lower levels of depression. Step 2 of the analysis introduces inequality perception as an additional predictor, which shows a significant positive relationship with depression (Coef. = 0.034, $p < 0.05$), indicating that higher levels of perceived inequality are associated with increased depression.

By Step 3, the interaction term between personal magnetism and inequality perception is introduced and is found to be significant (Coef. = -0.016, $p < 0.05$), denoting that the effect of personal magnetism on depression varies depending on the level of inequality perception. Specifically, personal magnetism's protective effect against depression is moderated by the perception of inequality, with a greater impact in contexts of lower inequality perception. Self-rated health remains a strong negative predictor of depression throughout all models (Coef. = -0.430, $p < 0.001$ in Step 3), reinforcing its importance as a determinant of mental well-being. These findings highlight the importance of socio-economic context in mental health and suggest that interventions aimed at reducing perceived inequality may enhance the effectiveness of strategies leveraging personal social resources to improve mental health outcomes.

Table 2. Multivariate regression results integrating inequality perception, CFPS 2018-2020 (N = 26,711).

Variable	Step 1 (DV: Depression)	Step 2 (DV: Depression)	Step 3 (DV: Depression)	Step 4 (DV: Personal magnetism)
Sex	-1.734	-1.728	-1.695	0.356
	(-1.54)	(-1.53)	(-1.50)	(0.62)
Age	0.034	0.041*	0.040	-0.018
	(1.69)	(1.98)	(1.95)	(-1.73)
Marital status	-0.258	-0.257	-0.257	0.069
	(-1.30)	(-1.29)	(-1.30)	(0.68)
Education	-0.069	-0.066	-0.067	-0.084
	(-0.53)	(-0.51)	(-0.51)	(-1.25)
Self-rated health	-0.429***	-0.429***	-0.430***	0.088***
	(-10.88)	(-10.90)	(-10.91)	(4.37)
Smoking	0.181	0.180	0.176	-0.036
	(1.05)	(1.05)	(1.02)	(-0.41)
Alcohol drinking	0.047	0.039	0.039	-0.060
	(0.36)	(0.29)	(0.30)	(-0.89)
Residence registration	0.015	0.015	0.013	-0.061

	(0.09)	(0.09)	(0.08)	(-0.73)
Personal magnetism	-0.113***	-0.116***	-0.003	
	(-5.07)	(-5.19)	(-0.06)	
Inequality perception		0.034*	0.146**	0.043***
		(2.08)	(2.76)	(5.16)
Interaction			-0.016*	
			(-2.22)	

t values in parentheses. * $p < 0.05$, ** $p < 0.01$, *** $p < 0.001$. Interaction = Personal magnetism * Inequality perception.

Table 3 details the effects of personal magnetism and community trust on depression. The results from Step 1 demonstrate a significant negative association between personal magnetism and depression (Coef. = -0.113, $p < 0.001$), suggesting that individuals with higher personal magnetism experience lower levels of depression. As community trust is introduced in Step 2, it shows a negative correlation with depression as well (Coef. = -0.053, $p < 0.01$), reinforcing the notion that trust plays a crucial role in mental health. Moreover, the interaction between personal magnetism and community trust introduced in Step 3 is not significant, indicating that the effect of personal magnetism on depression does not vary substantially at different levels of community trust within this sample.

Further insights are gleaned from the progression across the steps of the model. In

Step 4, when community trust serves as the dependent variable, personal magnetism is strongly positively correlated (Coef. = 0.181, $p < 0.001$), revealing that individuals with higher levels of personal magnetism tend to have greater trust within their community. Additionally, age and self-rated health emerge as significant factors. Age is negatively associated with community trust (Coef. = -0.059, $p < 0.001$), indicating that younger individuals tend to have higher community trust. In contrast, better self-rated health is associated with slightly higher levels of community trust (Coef. = 0.067, $p < 0.01$). These findings illuminate the complex web of relationships between personal attributes, perceived social capital, and mental health, offering a nuanced perspective on the protective factors that can buffer against depression.

Table 3. Multivariate regression results integrating community trust, CFPS 2018-2020 (N = 26,711).

**Variable**	**Step 1 (DV: Depression)**	**Step 2 (DV: Depression)**	**Step 3 (DV: Depression)**	**Step 4 (DV: Community trust)**
Sex	-1.734	-1.696	-1.715	0.714
	(-1.54)	(-1.50)	(-1.52)	(1.12)
Age	0.034	0.031	0.031	-0.059***
	(1.69)	(1.54)	(1.52)	(-5.15)
Marital status	-0.258	-0.246	-0.245	0.233*

	(-1.30)	(-1.24)	(-1.23)	(2.08)
Education	-0.069	-0.070	-0.068	-0.011
	(-0.53)	(-0.53)	(-0.52)	(-0.14)
Self-rated health	-0.429***	-0.425***	-0.425***	0.067**
	(-10.88)	(-10.79)	(-10.79)	(3.04)
Smoking	0.181	0.170	0.171	-0.210*
	(1.05)	(0.99)	(0.99)	(-2.17)
Alcohol drinking	0.047	0.048	0.047	0.020
	(0.36)	(0.36)	(0.36)	(0.28)
Residence registration	0.015	0.016	0.017	0.017
	(0.09)	(0.10)	(0.10)	(0.18)
Personal magnetism	-0.113***	-0.104***	-0.167**	0.181***
	(-5.07)	(-4.59)	(-2.97)	(14.41)
Community trust		-0.053**	-0.123*	
		(-2.60)	(-2.02)	
Interaction			0.010	
			(1.23)	

t values in parentheses. * $p < 0.05$, ** $p < 0.01$, *** $p < 0.001$. Interaction = Personal

magnetism * Community trust.

The moderating effect of inequality perception

Table 4 captures the moderating effect of inequality perception on the relationship between personal magnetism and mental health outcomes. The analysis provides a statistically significant negative coefficient for personal magnetism (Coef. = -0.118, $p < 0.001$), which highlights its potential role in mitigating adverse mental health effects. Inequality perception itself, while contributing a smaller positive coefficient (Coef. = 0.035, $p < 0.05$), suggests that higher perceptions of inequality correlate with poorer mental health outcomes. Moreover, the interaction term between personal magnetism and inequality perception is significant (Coef. = -0.016, $p < 0.05$), indicating that the impact of personal magnetism on mental health is conditional upon the level of perceived inequality within the society.

The model further demonstrates the intricacies of socio-demographic factors, as evidenced by the non-significant coefficients for sex, age, marital status, education, smoking, alcohol drinking, and residence registration, suggesting that these factors do not moderate the effect of personal magnetism and inequality perception on mental health in the same way. Significantly, self-rated health shows a substantial negative association with mental health outcomes (Coef. = -0.430, $p < 0.001$), reaffirming its status as a critical indicator of overall well-being. These findings underscore the nuanced complexities of psychological resilience in the face of socio-economic challenges and reinforce the importance of considering inequality perceptions in mental health assessments and interventions.

Table 4. The moderating role of inequality perception, CFPS 2018-2020 (N = 26,711).

Variable	Coef.	Std. Err.	t	p > |t|
C_Personal magnetism	-0.118***	0.022	-5.26	0.000
C_Inequality perception	0.035*	0.016	2.11	0.035
C_Interaction	-0.016*	0.007	-2.22	0.026
Sex	-1.695	1.127	-1.50	0.133
Age	0.040	0.021	1.95	0.051
Marital status	-0.257	0.198	-1.30	0.195
Education	-0.067	0.131	-0.51	0.608
Self-rated health	-0.430***	0.039	-10.91	0.000
Smoking	0.176	0.172	1.02	0.306
Alcohol drinking	0.039	0.131	0.30	0.766
Residence registration	0.013	0.163	0.08	0.934

* $p < 0.05$, ** $p < 0.01$, *** $p < 0.001$. C_Interaction = C_Personal magnetism * C_Inequality perception.

Figure 2 graphically represents the moderating effect of inequality perception on the relationship between personal magnetism and depression. The depicted trend lines

illustrate that individuals with lower personal magnetism scores tend to report higher levels of depression; however, this relationship is significantly moderated by the individuals' perception of inequality. For those with a lower perception of inequality, the decline in depression levels associated with increased personal magnetism is more pronounced (solid line), compared to individuals with a higher perception of inequality (dashed line), where personal magnetism seems to have a less substantial effect on reducing depression levels.

The figure elucidates a vital aspect of the social context's influence on mental health, revealing that the perceived fairness of the socio-economic environment can either buffer or exacerbate the impact of personal traits on psychological well-being. The slopes indicate a steeper decline in depression scores, moving from lower to higher personal magnetism at lower levels of perceived inequality, suggesting a potential protective role of perceived social fairness. In contrast, when inequality perception is high, the protective effect of personal magnetism is notably diminished, highlighting the importance of addressing perceptions of social inequality as part of comprehensive mental health interventions.

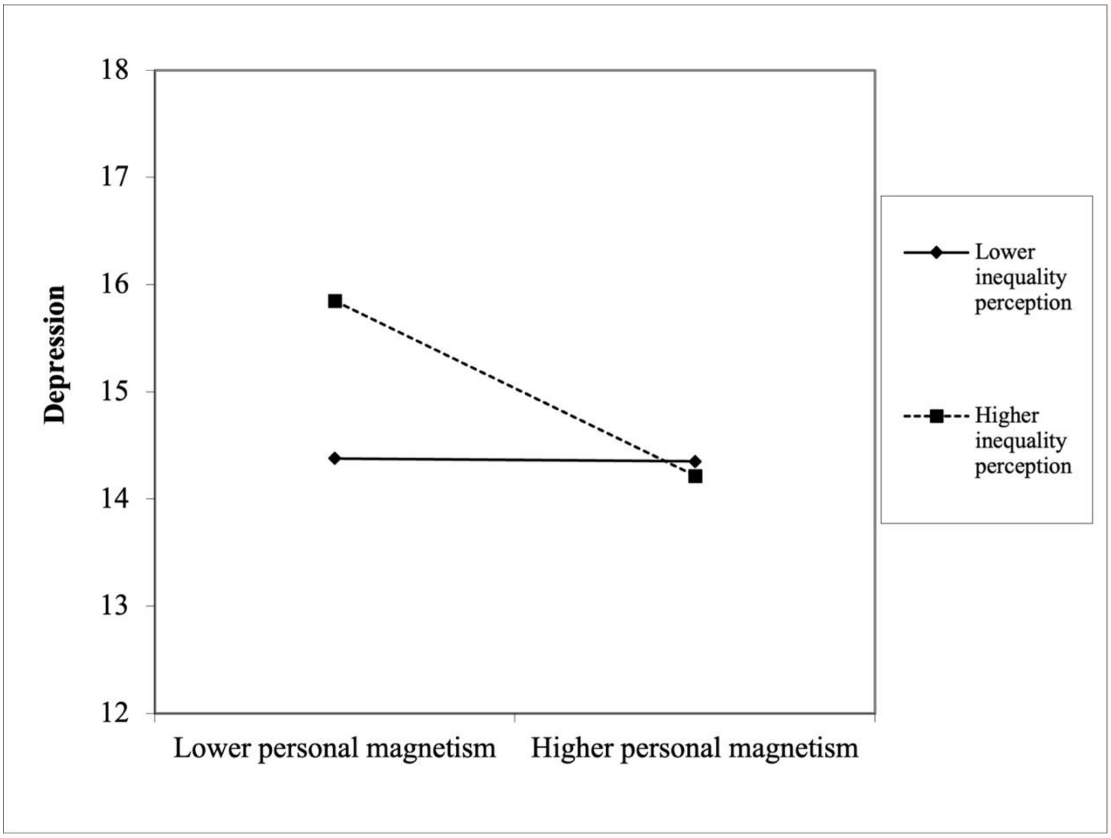

Figure 2. The moderating role of inequality perception.

Sex-based heterogeneity analysis

Table 5 elucidates the sex-based heterogeneity in the influence of personal magnetism, inequality perception, and their interaction on a range of variables. For females, personal magnetism exhibits a significant and robust inverse relationship with the dependent variable (Coef. = -0.175, $p < 0.001$), suggesting that personal magnetism may be a stronger protective factor against the negative outcomes measured, more so among females than males ($\beta = -0.063$, $p < 0.05$). Furthermore, the perception of inequality is positively associated with the dependent variable for females (Coef. = 0.061, $p < 0.05$), which is not observed in males (Coef. = 0.008, $p > 0.05$), indicating sex differences in how socio-economic perceptions impact outcomes.

Additionally, the interaction effect between personal magnetism and inequality perception is found to be non-significant for females (Coef. = -0.010, $p > 0.05$), whereas it presents as a significant factor for males (Coef. = -0.022, $p < 0.05$), highlighting that the protective effect of personal magnetism against adverse outcomes may diminish as inequality perception increases, especially in males. Notably, marital status shows a stark contrast between sexes, with a positive association for females (Coef. = 0.484, $p > 0.05$) and a highly significant negative association for males (Coef. = -0.921, $p < 0.001$), indicating that marital status plays differing roles in influencing outcomes across sexes. These findings underscore the need for nuanced approaches in addressing socioeconomic issues, taking into account the differential impacts of personal attributes and perceived societal structures based on sex.

Table 5. Sex-based heterogeneity test results, CFPS 2018-2020 (N = 26,711).

Variable	Female	Male
C_Personal magnetism	-0.175***<br>(-5.47)	-0.063*<br>(-2.01)
C_Inequality perception	0.061*<br>(2.54)	0.008<br>(0.37)
C_Interaction	-0.010<br>(-0.97)	-0.022*<br>(-2.23)

Age	0.038<br>(1.22)	0.042<br>(1.53)
Marital status	0.484<br>(1.69)	-0.921***<br>(-3.36)
Education	-0.228<br>(-1.21)	0.086<br>(0.47)
Self-rated health	-0.488***<br>(-8.81)	-0.372***<br>(-6.64)
Smoking	1.456*<br>(2.21)	0.132<br>(0.75)
Alcohol drinking	0.179<br>(0.46)	0.027<br>(0.20)
Residence registration	-0.073<br>(-0.34)	0.172<br>(0.69)

* $p < 0.05$, ** $p < 0.01$, *** $p < 0.001$. C_Interaction = C_Personal magnetism * C_Inequality perception.

The mediating effect of community trust

Table 6 presents a compelling examination of community trust's mediating role. The

total effect of the independent variable on depression, denoted by path c, was found to be significant and negative (Effect = -0.276, $p < 0.001$, 95% CI: -0.300, -0.252), indicating a robust inverse relationship. When accounting for the mediating effect of community trust, the direct effect, path c', remained negative (Effect = -0.213, $p < 0.001$, 95% CI: -0.240, -0.185), albeit reduced in magnitude. This reduction illustrates that while the independent variable exerts a direct influence on depression, community trust plays a substantial role in this association, with a significant indirect effect (Effect = -0.063, $p < 0.001$, 95% CI: -0.071, -0.056). The ratio of the indirect effect to the total effect (0.229) confirms that nearly 23% of the effect of the independent variable on depression is mediated by community trust.

Furthermore, the analysis delineates that the ratio of the indirect effect to the direct effect is approximately 0.298, suggesting that for every unit change in the independent variable, there is nearly a 30% change in depression that is accounted for by the mediating influence of community trust. This finding accentuates the protective nature of community trust against depression and reinforces the need to consider community-level interventions in mental health strategies. These significant mediating relationships provide evidence that strengthening community trust could be an effective lever for reducing the prevalence of depression in the population, thereby contributing to the literature advocating for the promotion of social capital as a public health resource.

Table 6. The mediating role of community trust, CFPS 2018-2020 (N = 26,711).

	**Effect**	**Std. Err.**	**LLCI**	**ULCI**	**Ratio**

Total effect (c)	-0.276	0.012	-0.300	-0.252	
Direct effect (c’)	-0.213	0.014	-0.240	-0.185	
Indirect effect (ab)	-0.063	0.004	-0.071	-0.056	
Ratio of indirect to total effect mediated					0.229
Ratio of indirect to direct effect					0.298

Note: The ‘c’ path denotes the direct effect of the independent variable on the dependent variable. The ‘ab’ path indicates the indirect effect of the independent variable on the dependent variable through the mediator. Conversely, the ‘c’ path delineates the effect of the independent variable on the dependent variable when accounting for the mediating variable.

Abbreviations: LLCI, Lower Limit of Confidence Interval; ULCI, Upper Limit of Confidence Interval.

Robustness test

To affirm the reliability of the findings, this study utilized three distinct approaches for the robustness analysis. First, the sample was modified by omitting individuals over 60 years of age, acknowledging that the evaluation of depression may diverge for older adults due to physiological changes and the transition to retirement [44, 45]. The outcomes remained consistent with those obtained from the complete sample. Second,

the original multivariate fixed-effects regression model was replaced with a fixed-effects ordered logit model [46], which upheld the robustness of the results. Third, an essential control variable, trust in strangers, was integrated into the model, scoring from 0 (indicating very low trust) to 10 (signifying very high trust). Given the study's focus on the nexus between personal magnetism and depression and considering that personal magnetism-related interpersonal disorders often precipitate depression, the inclusion of trust in strangers—a salient component in forming positive social interactions—was deemed imperative [47]. The incorporation of this variable did not alter the findings, further substantiating the robustness of the results.

Discussion

This study elucidates the intricate interrelation between personal magnetism and depression, unearthing a protective linkage consistent across various age groups within the Chinese populace. The findings resonate with the Confucian valorization of societal concord, positing that the presence of personal magnetism potentially acts as a bulwark against depressive symptoms by fortifying self-perception and fulfillment [48, 49]. By transcending the confines of adolescent-focused research, this study enhances the comprehensive understanding of such dynamics, thereby extending their relevance and applicability across a broader demographic spectrum.

More importantly, this study presents a nuanced examination of how perceived income inequality modulates the inverse relationship between personal magnetism and depression. It substantiates the principles of inequity aversion theory, proposing that an acute consciousness of economic disparities catalyzes a shift toward prosocial activities.

Such behaviors are conducive to the alleviation of depressive symptoms, as they foster community cohesion and individual well-being [50]. Within the socio-cultural fabric of China, where the quest for shared prosperity is deeply rooted, the cognizance of income gaps may invoke a collective empathetic response. This, in turn, may enhance the luster of personal magnetism, serving to strengthen the social bonds that are integral to individual and communal resilience [51]. This insight illuminates the potential of social awareness to not only bridge economic divides but also to forge psychological support networks that counteract the malaise of depression.

In this study, community trust is identified as a pivotal mediator, elucidating the mechanisms by which personal magnetism attenuates depressive symptoms. The study posits that individuals with higher personal magnetism are predisposed to cultivate expansive social networks, which in turn nurture a sense of community trust. This trust acts as a psychological scaffold, diminishing the prevalence and severity of depressive symptoms [52]. The significance of this finding is accentuated in the context of China's societal evolution from a 'Danwei'-centric to a community-oriented framework. In this new paradigm, community trust transcends mere social lubricant to become a fundamental tenet of societal engagement, with profound implications for the mental health and overall well-being of individuals [53]. The implication is that trust within community structures does not merely reflect social harmony but actively participates in the construction of a supportive environment conducive to psychological health. This underscores the necessity for policy interventions that prioritize the restoration and cultivation of trust at the community level as a strategic approach to combat depression

in transitional societies.

The theoretical enrichment offered by this study underscores the dynamic interplay between individual attributes and socio-economic factors in shaping mental health outcomes. It encourages a shift in public health strategies towards more holistic and socially integrated approaches to mental health care, particularly within the rapidly transforming societal landscapes of urbanizing regions.

By advocating for policies that not only acknowledge but actively address income inequality, there is potential to catalyze prosocial behavior and fortify communal bonds, thereby augmenting individual resilience to depressive symptoms. In parallel, initiatives aimed at restoring trust within communities could underpin the development of mental health interventions that emphasize collective engagement and mutual support. Incorporating these insights into the practice of social work necessitates a multidimensional approach that integrates economic, social, and psychological considerations. Social workers, operating at the nexus of individual experiences and societal structures, are uniquely positioned to spearhead community-level interventions that bolster personal magnetism through trust-building activities, advocacy for equitable resource distribution, and the promotion of inclusive community practices.

Conclusion

This study elucidates the intricate association between personal magnetism and depression, shedding light on the mediating and moderating roles of community trust and perceived income inequality within the context of China's evolving societal structure. Markedly, this study pioneers the exploration of these nuanced interactions,

providing empirical substantiation for the moderating influence of perceived income inequality on the depression-personal magnetism continuum. This aligns with inequity aversion theory, emphasizing the imperative to elevate societal awareness of social disparities, thereby nurturing prosocial behaviors that bolster personal magnetism.

Moreover, the revelation that community trust acts as a mediator in this relationship heralds important implications for mental health policy and community-oriented social strategies. The findings advocate for policy reforms that prioritize the reinforcement of community trust, recognizing that the cultivation of such trust can significantly magnify the positive impact of personal magnetism on mental health. By advocating for a culture of mutual support and collective resilience, such policy initiatives can contribute to the amelioration of mental health outcomes in transitional societies, emphasizing the role of social capital as a cornerstone of public well-being.

References

1. Urdinez F, Masiero G. China and the WTO: Will the market economy status make any difference after 2016. Chin Econ. 2015; 48(2): 155-172.

2. Wu D, Rao P. Urbanization and income inequality in China: An empirical investigation at provincial level. Soc Indic Res. 2017; 131(1): 189-214.

3. Huang Y, Wang YU, Wang H, et al. Prevalence of mental disorders in China: A cross-sectional epidemiological study. Lancet Psychiatry. 2019; 6(3): 211-224.

4. Sullivan HS. The interpersonal theory of psychiatry. New York: Norton, 1953.

5. Baumeister RF, Leary MR. The need to belong: Desire for interpersonal attachments as a fundamental human motivation. Psychol Bull. 1995; 117(3): 497-529.

6. Kawachi I, Berkman L. Social ties and mental health. J Urban Health. 2001; 78(3): 458-467.

7. Cacioppo JT, Hawkley LC. Social isolation and health, with an emphasis on underlying mechanisms. Perspect Biol Med. 2003; 46(S3): S39-S52.

8. Whyte MK. The Chinese family and economic development: Obstacle or engine? Econ Dev Cult Change. 1996; 45(1): 1-30.

9. Li L, Logan JR. The transformation of urban China. J Chin Sociol. 2018; 5(1): 1-11.

10. Chen F. Between the state and labour: The conflict of Chinese trade unions' double identity in market reform. China Q. 2003; 176: 1006-1028.

11. Putnam RD. Bowling alone: The collapse and revival of American community. New York, NY: Simon and Schuster, 2000.

12. Helliwell JF, Putnam RD. The social context of well-being. Philos Trans R Soc Lond B Biol Sci. 2004; 359(1449): 1435-1446.

13. Fehr E, Schmidt KM. A theory of fairness, competition, and cooperation. Q J Econ. 1999; 114(3): 817-868.

14. Engelmann D, Strobel M. Inequality aversion, efficiency, and maximin preferences in simple distribution experiments. Am Econ Rev. 2004; 94(4): 857-869.

15. Adams JS. Inequity in social exchange. In: Berkowitz L, ed. Advances in experimental social psychology. Cambridge, MA: Academic Press, 1965; Vol. 2: 267-299.

16. Tyler TR, Blader SL. The group engagement model: Procedural justice, social identity, and cooperative behavior. Pers Soc Psychol Rev. 2003; 7(4): 349-361.

17. Crosby F. A model of egoistical relative deprivation. Psychol Rev. 1976; 83(2): 85-113.

18. Smith HJ, Pettigrew TF, Pippin GM, Bialosiewicz S. Relative deprivation: A theoretical and meta-analytic review. Pers Soc Psychol Rev. 2012; 16(3): 203-232.

19. Portes A. Social capital: Its origins and applications in modern sociology. Annu Rev Sociol. 1998; 24: 1-24.

20. Kawachi I, Kennedy BP, Glass R. Social capital and self-rated health: A contextual analysis. Am J Public Health. 1999; 89(8): 1187-1193.

21. Festinger L. A theory of social comparison processes. Human Relations. 1954; 7(2): 117-140.

22. Wilkinson R, Pickett K. The spirit level: Why greater equality makes societies stronger. New York, NY: Bloomsbury Press, 2010.

23. Diener E, Biswas-Diener R. Will money increase subjective well-being? A literature review and guide to needed research. Soc Indic Res. 2002; 57(2): 119-169.

24. Layte R. The association between income inequality and mental health: Testing status anxiety, social capital, and neo-materialist explanations. Eur Sociol Rev. 2012; 28(4): 498-511.

25. Uslaner EM. The moral foundations of trust. Cambridge: Cambridge University Press, 2002.

26. De Silva MJ, McKenzie K, Harpham T, Huttly SR. Social capital and mental illness: A systematic review. J Epidemiol Community Health. 2005; 59(8): 619-627.

27. Woolcock M, Narayan D. Social capital: Implications for development theory,

research, and policy. World Bank Res Obs. 2000; 15(2): 225-249.

28. Cohen S, Wills TA. Stress, social support, and the buffering hypothesis. Psychol Bull. 1985; 98(2): 310-357.

29. Thoits PA. Mechanisms linking social ties and support to physical and mental health. J Health Soc Behav. 2011; 52(2): 145-161.

30. Berkman LF, Glass T, Brissette I, Seeman TE. From social integration to health: Durkheim in the new millennium. Soc Sci Med. 2000; 51(6): 843-857.

31. Szreter S, Woolcock M. Health by association? Social capital, social theory, and the political economy of public health. Int J Epidemiol. 2004; 33(4): 650-667.

32. Rose R. How much does social capital add to individual health? A survey study of Russians. Soc Sci Med. 2000; 51(9): 1421-1435.

33. Radloff LS. The CES-D scale: A self-report depression scale for research in the general population. Appl Psychol Meas. 1977; 1(3): 385-401.

34. Yu SC, Lin YH, Hsu WH. Applying structural equation modeling to report psychometric properties of Chinese version 10-item CES-D depression scale. Qual Quant. 2013; 47(3): 1511-1518.

35. Burt A. A mechanistic explanation of popularity: Genes, rule breaking, and evocative gene–environment correlations. J Pers Soc Psychol. 2009; 96(4): 783-794.

36. Ellis WE, Dumas TM, Hutchinson LR, Talebi S. Staying safe or staying popular? Popularity and reputation concerns predict adherence and adjustment during the COVID-19 pandemic. Youth Soc. 2022; 55(7): 1287-1306.

37. Kim H, Huh S, Choi S, Lee Y. Perceptions of inequality and attitudes towards

redistribution in four East Asian welfare states. Int J Soc Welfare. 2018; 27(1): 28-39.

38. Lundåsen SW. Intergroup contacts, neighborhood diversity, and community trust: The asymmetrical impact of negative and positive experiences. J Int Migr Integr. 2022; 24(1): 163-188.

39. Li N, Zhang J. Influencing factors for depression among Chinese suicide survivors. Psychiatry Res. 2010; 178(1): 97-100.

40. Lu S, Reavley N, Zhou J, et al. Depression among the general adult population in Jiangsu Province of China: Prevalence, associated factors and impacts. Soc Psychiatry Psychiatr Epidemiol. 2018; 53(10): 1051-1061.

41. Gavin MB, Hofmann DA. Using hierarchical linear modeling to investigate the moderating influence of leadership climate. Leadersh Q. 2002; 13(1): 15-33.

42. Busenbark JR, Graffin SD, Campbell RJ, Lee EY. A marginal effects approach to interpreting main effects and moderation. Organ Res Methods. 2022; 25(1): 147-169.

43. Zhao X, Lynch Jr JG, Chen Q. Reconsidering Baron and Kenny: Myths and truths about mediation analysis. J Consum Res. 2010; 37(2): 197-206.

44. Jokelainen J, Timonen M, Keinänen-Kiukaanniemi S, et al. Growth and common prosperity in China. China World Econ. 2022; 30(1): 28-57.

45. Krishnamoorthy Y, Rajaa S, Rehman T. Diagnostic accuracy of various forms of geriatric depression scale for screening of depression among older adults: Systematic review and meta-analysis. Arch Gerontol Geriatr. 2020; 87: 104002.

46. Muris C. Estimation in the fixed-effects ordered logit model. Rev Econ Stat. 2017; 99(3): 465-477.

47. Ueno K. The effects of friendship networks on adolescent depressive symptoms. Soc Sci Res. 2005; 34(3): 484-510.

48. Cao W, Li L, Zhou X, Zhou C. Social capital and depression: Evidence from urban elderly in China. Aging Ment Health. 2015; 19(5): 418-429.

49. Stankov L. Depression and life satisfaction among European and Confucian adolescents. Psychol Assess. 2013; 25(4): 1220-1234.

50. Alarcón G, Forbes EE. Prosocial behavior and depression: A case for developmental gender differences. Curr, Behav. Neurosci Rep. 2017; 4(2): 117-127.

51. Newman BJ, Hartman TK, Lown PL, Feldman S. Easing the heavy hand: Humanitarian concern, empathy, and opinion on immigration. Br J Polit Sci. 2015; 45(3): 583-607.

52. Wu Z, Zhao R, Zhang X, Liu F. The impact of social capital on volunteering and giving: Evidence from urban China. Nonprofit Volunt Sect Q. 2018; 47(6): 1201-1222.

53. Stolle D, Soroka S, Johnston R. When does diversity erode trust? Neighborhood diversity, interpersonal trust and the mediating effect of social interactions. Polit Stud. 2008; 56(1): 57-75.
