# Supplementary material for: Nesting of groups: Links among personal magnetism, community trust, inequality perception and depression
Source: PLoS One. 2025 Mar 25;20(3):e0320560. doi: 10.1371/journal.pone.0320560 (PMC11936175; doi:10.1371/journal.pone.0320560)
Supplement: Attachment — Submitted filename: 0217 Revised Response Letter.docx [file pone.0320560.s003.docx]

Dear Editor and Reviewers,

We would like to sincerely thank you for your valuable feedback on our manuscript entitled “Nesting of groups: Links among personal magnetism, community trust, inequality perception, and depression.” We greatly appreciate the time and effort you have taken to review our work, and your thoughtful suggestions have played a crucial role in enhancing the quality and clarity of the paper.

In response to the editorial and reviewer comments, we have made comprehensive revisions to the manuscript. Specifically, we have addressed the following points:

**1. Justification of the relevance of the phenomenon**

We added a paragraph at the end of the Introduction to emphasize the importance of the study in the context of China’s socio-economic transformation and its implications for mental health, particularly regarding the interplay between personal magnetism, community trust, and inequality perception.

**2. Update of the literature review**

We expanded the literature review, particularly regarding interpersonal trust, to include more recent studies linking community trust to mental well-being, especially in transitional societies like China.

**3. Practical implications**

A new section was added to the Conclusion, detailing the role of institutions in promoting community trust as a strategy to enhance social cohesion, well-being, and reduce the psychological burden of inequality.

**4. Clarification of the age range in the sample**

We have provided additional clarification regarding the age range of the participants, focusing on the age segmentation of individuals aged 16-59 and why this cohort was chosen for the robustness check.

**5. Revision of wording**

We have carefully revised the wording to enhance clarity and address the concerns related to phrasing.

We have also proofread the manuscript for grammatical accuracy and made structural adjustments to improve the flow of the paper. All changes have been clearly marked in the revised manuscript for your review.

We hope these revisions have strengthened the manuscript, making it more comprehensive, transparent, and aligned with the feedback provided. We are grateful for the opportunity to submit our revised manuscript for further consideration.

Thank you for your time and consideration.

Sincerely yours.

**Response to Editor**

**Point 1:** Thank you for submitting your manuscript to PLOS ONE. After careful consideration, we feel that it has merit but does not fully meet PLOS ONE’s publication criteria as it currently stands. Therefore, we invite you to submit a revised version of the manuscript that addresses the points raised during the review process.

Dear author, please review the comments made, particularly those submitted by the second reviewer, and make the changes following the instructions below. It is necessary that you clearly identify the changes made and their place in the text. Additionally, it is important that you update the literature review regarding interpersonal trust in the context under study. Additionally, it is important that in the first section of the manuscript, just before the end, you justify in a paragraph why the phenomenon investigated in the research is relevant. Finally, in the final section, it is necessary to include a brief section showing the practical implications of the research in relation to public policies. Specifically, it is important to describe the role that institutions play in promoting (social) trust among community members as a strategy to promote the well-being and development of society.

Please submit your revised manuscript by Mar 26 2025 11:59PM. If you will need more time than this to complete your revisions, please reply to this message or contact the journal office at plosone@plos.org. When you’re ready to submit your revision, log on to https://www.editorialmanager.com/pone/ and select the ‘Submissions Needing Revision’ folder to locate your manuscript file.

Please include the following items when submitting your revised manuscript:

•	A rebuttal letter that responds to each point raised by the academic editor and reviewer(s). You should upload this letter as a separate file labeled ‘Response to Reviewers’.

•	A marked-up copy of your manuscript that highlights changes made to the original version. You should upload this as a separate file labeled ‘Revised Manuscript with Track Changes’.

•	An unmarked version of your revised paper without tracked changes. You should upload this as a separate file labeled ‘Manuscript’.

If you would like to make changes to your financial disclosure, please include your updated statement in your cover letter. Guidelines for resubmitting your figure files are available below the reviewer comments at the end of this letter.

If applicable, we recommend that you deposit your laboratory protocols in protocols.io to enhance the reproducibility of your results. Protocols.io assigns your protocol its own identifier (DOI) so that it can be cited independently in the future. For instructions see: https://journals.plos.org/plosone/s/submission-guidelines#loc-laboratory-protocols. Additionally, PLOS ONE offers an option for publishing peer-reviewed Lab Protocol articles, which describe protocols hosted on protocols.io. Read more information on sharing protocols at https://plos.org/protocols?utm_medium=editorial-email&utm_source=authorletters&utm_campaign=protocols.

We look forward to receiving your revised manuscript.

Kind regards,

PLOS ONE

**Response 1:**

Dear Editor,

Thank you for your thoughtful feedback and for providing us with the opportunity to revise our manuscript. We appreciate the valuable insights provided by both the reviewers and yourself. We have carefully addressed all the points raised in your review and made the necessary revisions to enhance the clarity, depth, and overall quality of the manuscript. Below, we provide a detailed response to each of the points mentioned.

**1. Justification of the relevance of the phenomenon investigated in the research**

In response to your comment about providing a clearer justification for the relevance of the research, we have added a paragraph at the end of the Introduction section. This new addition emphasizes the critical importance of understanding the interplay between personal magnetism, community trust, and inequality perception in the context of rapid socio-economic changes in China. We explain that this study is particularly relevant as China’s transition from a collectivist work-unit system to a more individualized, community-oriented society has led to an erosion of traditional communal networks, contributing to increased feelings of isolation and mental distress. The relevance of our study lies in addressing how personal magnetism can serve as a buffer against these negative mental health outcomes and how community trust plays a crucial mediating role in this process, especially in a society grappling with heightened income inequality.

**The revised part is as follows:**

**Introduction**

China’s accession to the World Trade Organization (WTO) marked the onset of an unprecedented economic expansion, catalyzing a profound restructuring of its social fabric [1]. This rapid economic transformation has deepened socioeconomic stratification, eroding communal networks that historically functioned as pillars of social trust and collective identity. As traditional support systems weaken, individuals face diminished access to trust-based social capital, contributing to a fragmentation of interpersonal relationships and a decline in community cohesion [2]. These structural shifts have significant mental health implications, as evidenced by the 6.8% lifetime prevalence of depressive disorders among Chinese adults, alongside persistently low treatment engagement rates, highlighting the urgency of addressing the psychosocial consequences of rapid societal change [3].

By examining the interplay between personal magnetism, community trust, inequality perception, and depression, this study provides critical insights into the mechanisms through which individuals navigate social disembedding and adapt to transforming trust structures. More broadly, this study advances an empirically grounded framework for understanding the role of social trust and interpersonal networks in fostering psychological resilience. Its findings offer policy-relevant strategies for rebuilding social cohesion, reducing mental health disparities, and strengthening collective well-being in societies undergoing profound structural transformation.

**2. Literature review regarding interpersonal trust in the context under study**

In line with your suggestion, we have significantly updated the Literature Review to provide a more detailed exploration of interpersonal trust, particularly in the context of socio-economic transformations and mental health. This revision draws on recent studies that link community trust with mental well-being, highlighting its role as a critical psychosocial resource in transitional societies like China. We specifically discuss how interpersonal trust networks have been shown to buffer against depressive symptoms, especially when formal mental health services are scarce or stigmatized. We also emphasize that trust not only enhances social cohesion but also supports psychological resilience in individuals who may feel marginalized by societal changes.

**The revised part is as follows:**

**Literature review**

**Personal magnetism and depression**

The construct of personal magnetism occupies a critical position in psychological and social theory, particularly in its role as a protective factor against depressive symptoms. Grounded in social psychology, personal magnetism is conceptualized as an individual’s capacity to attract and maintain social relationships, fostering strong interpersonal connections and social integration. These connections extend beyond superficial interactions, serving as psychosocial resources that reinforce self-worth, provide emotional support, and enhance resilience against adversity [4, 5].

Empirical research has substantiated the protective effects of personal magnetism on mental health, emphasizing the pivotal role of social bonds in mitigating psychological distress. For instance, Kawachi and Berkman assert that the breadth and depth of an individual’s social networks—core components of personal magnetism—buffer against stressors that contribute to depression [6]. Similarly, Cacioppo and Hawkley identify loneliness as the antithesis of social magnetism, demonstrating its strong association with depressive symptomatology, thereby underscoring the importance of social connectedness in mental health regulation [7].

In transitional societies such as China, where rapid socio-economic changes have eroded traditional social structures, personal magnetism assumes an even greater adaptive function. The dissolution of the work-unit (Danwei) system, which historically provided institutionalized social stability through workplace-based communities, has resulted in a fragmentation of trust networks, creating a void that personal magnetism can effectively bridge [8, 9]. This structural transformation necessitates new mechanisms of social integration, positioning personal magnetism as a critical asset in the reconstruction of interpersonal networks and the preservation of psychological well-being [10].

Given that individuals with greater personal magnetism are more likely to establish and sustain meaningful social relationships, which in turn function as buffers against psychological distress [11, 12], this study hypothesizes:

**Hypothesis 1:** Personal magnetism is negatively correlated with depression.

**The moderating role of perceived inequality**

The relationship between personal magnetism and depression is not uniform across social contexts but is contingent upon individuals’ perceptions of income inequality. Perceived inequality serves as a moderator, shaping how personal magnetism influences mental health by altering individuals’ social comparisons, expectations of fairness, and access to psychosocial resources. The moderating role of perceived inequality can be understood through the lens of inequity aversion theory, which suggests that individuals who perceive greater economic disparities may experience heightened psychological distress, as such perceptions often generate feelings of social injustice and status anxiety [13, 14]. In this context, even individuals with high personal magnetism may struggle to leverage their social networks as effective protective mechanisms, as widespread perceptions of inequality can erode trust, intensify competition for resources, and amplify the psychological burden associated with social standing [15, 16].

Furthermore, relative deprivation theory provides additional insight into how perceived inequality exacerbates the relationship between personal magnetism and depression. Relative deprivation occurs when individuals feel disadvantaged relative to their peers, fostering frustration, alienation, and lower psychological well-being [17, 18]. Individuals with high personal magnetism often engage in frequent social comparisons, as their social standing is tied to peer validation and interpersonal influence. However, in environments characterized by high perceived inequality, upward social comparisons may become more distressing, leading to diminished self-worth and increased vulnerability to depressive symptoms. In contrast, in societies with lower perceived inequality, social networks function more as sources of emotional support and collective identity, allowing personal magnetism to maintain its psychological buffering effects [19, 20].

Social comparison theory further elucidates this moderating role by emphasizing that individuals constantly evaluate their socio-economic position relative to others [21]. In contexts where inequality perception is low, individuals with high personal magnetism may derive greater psychological benefits from their social interactions, as they experience higher levels of trust and reciprocity within their networks. However, in environments of high inequality perception, these social interactions may become more hierarchical, competitive, and psychologically taxing, weakening the protective function of personal magnetism. The persistent awareness of economic disparities can intensify status anxiety and reinforce the negative effects of unfavorable comparisons, making individuals with high personal magnetism even more susceptible to depression [22].

Taken together, these theoretical perspectives suggest that perceived inequality does not neutralize the relationship between personal magnetism and depression but rather strengthens it. As perceptions of inequality intensify, the social and psychological costs of maintaining high personal magnetism increase, amplifying stress and diminishing the emotional benefits typically associated with strong interpersonal ties [23, 24]. This insight highlights the interactive nature of individual social capital and structural economic conditions, revealing that personal magnetism may become a double-edged sword in highly stratified social environments. Thus, this study proposes:

**Hypothesis 2:** Inequality perception strengthens the relationship between personal magnetism and depression.

**The mediating role of community trust**

Community trust, conceptualized as the collective expectation of fairness, reciprocity, and social reliability, serves as a fundamental mechanism through which interpersonal dynamics influence psychological well-being [25]. As societies undergo structural transformations, traditional forms of social embeddedness are increasingly replaced by individualized and transactional relationships, leading to the fragmentation of social trust networks [26]. This erosion of trust weakens informal social support systems, exacerbating feelings of social isolation and psychological distress, which in turn heighten the risk of depression [27]. Within this evolving social landscape, personal magnetism—an individual’s ability to form and maintain strong interpersonal connections—plays a crucial role in rebuilding trust structures. Individuals with high personal magnetism are more likely to facilitate trust-based interactions, enhance social reciprocity, and contribute to the reinforcement of collective norms, thereby strengthening community trust and fostering psychological resilience [4, 5].

The mediating role of community trust in the personal magnetism-depression relationship is particularly salient in contexts where mental health stigma is pervasive and access to formal psychological interventions is limited [3]. Extensive research suggests that individuals embedded in high-trust communities report lower levels of stress, anxiety, and depressive symptoms, as trust functions as a critical form of social capital that enhances collective efficacy, promotes mutual aid, and reduces social uncertainty [30]. Trust-based networks foster a sense of belonging and inclusion, enabling individuals to access both emotional and instrumental support, which in turn buffers against psychological distress and mitigates the risk of depression [28]. Given that China’s mental health infrastructure remains underdeveloped, informal trust networks often act as functional substitutes for institutional support systems, making community trust a key determinant of mental health outcomes, particularly for individuals who experience social or economic precarity [8, 9].

The mediating effect of community trust in the personal magnetism-depression relationship operates through three primary pathways. First, individuals with high personal magnetism engage in frequent reciprocal social interactions, reinforcing perceptions of reliability and trustworthiness, which strengthen social bonds and collective trust norms [29]. Second, highly magnetic individuals often occupy central positions in social networks, acting as bridges that facilitate trust diffusion and foster group cohesion [30]. Third, trust cultivated at the interpersonal level spills over into broader community contexts, reinforcing generalized trust norms that extend beyond immediate social circles, thereby amplifying the protective effects of trust on mental health resilience [31, 32]. Thus, this study proposes:

**Hypothesis 3:** Community trust mediates the relationship between personal magnetism and depression.

By positioning community trust as a mediating mechanism, this study provides a conceptual framework for understanding how individual social traits translate into broader structural processes, thereby bridging the micro-macro divide in trust and mental health research. Figure 1 visually represents the proposed research framework.

**Figure 1.** The conceptual framework of the relationship between personal magnetism and depression.

**References**

25. Wu Y, Krueger F. Charting the neuroscience of interpersonal trust: A bibliographic literature review. Neurosci Biobehav Rev. 2024; 105930.

26. Okoye D. Things fall apart? Missions, institutions, and interpersonal trust. J Dev Econ. 2021; 148: 102568.

27. Liu N, Bao G, Wu S. Social implications of Covid-19: Its impact on general trust, political trust, and trust in physicians in China. Soc Sci Med. 2023; 317: 115629.

28. He AJ. The welfare is ours: Rural-to-urban migration and domestic welfare chauvinism in urban China. J Contemp China. 2022; 31(134): 202-218.

29. Chen X, Liu Y, Zhong H. Generalized trust among rural-to-urban migrants in China: Role of relative deprivation and neighborhood context. Int J Intercult Relat. 2023; 94: 101784.

30. Kim SH. Love thy neighbor? The role of trust in neighbors during the COVID-19 crisis. Health Place. 2024; 85: 103164.

31. Mao S, Lu N, Xiao C. Perceived neighborhood environment and depressive symptoms among older adults living in Urban China: The mediator role of social capital. Health Soc Care Community. 2022; 30(5): e1977-e1990.

32. Jia Z, Xu S, Zhang Z, Cheng Z, Han H, Xu H, Zhou Z. Association between mental health and community support in lockdown communities during the COVID-19 pandemic: Evidence from rural China. J Rural Stud. 2021; 82: 87-97.

**3. Practical implications of the research in relation to public policies**

We have included a new section in the Conclusion that addresses the practical implications of our findings in the context of public policy. This addition specifically highlights the role of institutions in fostering community trust as a strategy to promote societal well-being and individual mental health. We propose that government bodies, non-governmental organizations, and community-based institutions have a key role in creating trust-based social capital. In particular, we emphasize the importance of policies that promote transparent governance, economic equity, and community engagement in decision-making processes. By addressing structural inequality and promoting social solidarity, these policies can strengthen community networks and reduce the psychological burden of inequality, which in turn can mitigate depression.

**The revised part is as follows:**

**Conclusion**

This study enhances our understanding of mental health by conceptualizing depression as a complex, socially embedded phenomenon, influenced by both individual psychological traits and broader socio-structural factors. By examining the interaction between personal magnetism, community trust, and perceived inequality, the study introduces a multidimensional approach that recognizes the critical role of social structures in shaping mental health outcomes. The findings challenge traditional models of mental health that focus primarily on individual-level factors and emphasize the importance of understanding mental well-being as a dynamic interplay between individual agency and social context.

From a policy perspective, the study highlights the central role of institutions in fostering community trust as a strategy for improving mental health and well-being. Given that community trust mediates the relationship between personal magnetism and mental health outcomes, institutions—especially local governments, non-governmental organizations, and community-based networks—must prioritize the cultivation of trust-based social capital. This involves creating an environment that encourages social cooperation, transparency, and mutual respect, while also addressing the material conditions of inequality through equitable resource distribution. Policies that promote inclusive economic opportunities, universal access to public services, and targeted social welfare programs can help mitigate the adverse effects of inequality on mental health, fostering a more cohesive and resilient society.

Furthermore, policies should aim to strengthen participatory governance structures, where community members are actively engaged in decision-making processes. Such initiatives can foster collective efficacy and social cohesion, further enhancing community trust and reducing the psychological burden of inequality. By addressing both the structural and relational dimensions of social life, these policies can create a more supportive environment for mental health and well-being.

Ultimately, the findings suggest that mental health policy must evolve beyond an individualistic focus to incorporate the social and structural determinants of well-being. By emphasizing community trust and reducing inequality, policymakers can build a more resilient, psychologically stable society. Integrating these social determinants into public health strategies offers a holistic approach to mental health, ensuring that interventions are not only effective but also sustainable in the long term. This study calls for a paradigm shift in mental health policy—one that recognizes the inseparable links between individual mental health, social capital, and socio-economic conditions, and that prioritizes the creation of trustful, inclusive, and supportive communities.

**4. Proofreading and structural adjustments**

First, we have carefully proofread the entire manuscript to improve readability and clarity, addressing minor grammatical errors and inconsistencies in wording. This has ensured that the language is clearer, more concise, and professionally presented.

Second, we have revised the Discussion section to enhance the depth of analysis and provide a more nuanced interpretation of the results. Specifically, we have clarified the implications of our findings on personal magnetism, community trust, and perceived inequality, making stronger connections between theory and empirical results.

**The revised part is as follows:**

**Discussion**

This study contributes to the growing body of research on mental health by advancing our understanding of the intricate, multi-layered relationships between personal magnetism, perceived inequality, community trust, and depression within a rapidly transforming socio-economic context. Specifically, it sheds light on how these factors interact to shape mental health outcomes, offering valuable insights into the mechanisms by which social capital and structural inequality converge with individual psychological traits to influence depression. By framing depression as a socially embedded phenomenon, this study moves beyond individualistic explanations and highlights the importance of considering broader socio-structural dynamics.

The findings regarding personal magnetism’s role in protecting against depression align with existing theories of social capital, suggesting that individuals with higher social appeal and the ability to forge extensive interpersonal networks experience lower levels of depression [48, 49]. However, it is critical to recognize that this protective effect is not solely an outcome of individual charisma or charm, but rather the result of broader social processes that enable individuals to leverage their social capital. In transitional societies like China, where rapid urbanization and market reforms disrupt traditional social structures, personal magnetism serves as a vital tool for navigating the process of social disembedding, which refers to the erosion of stable, long-term social ties [1-3]. By facilitating the formation of new, meaningful relationships, personal magnetism plays a key role in fostering a sense of belonging and stability in environments marked by social flux. This highlights personal magnetism not only as a psychological trait but also as a form of social capital that is especially critical in contexts where formal mental health support structures are underdeveloped.

While personal magnetism serves as a protective buffer against depression, the study also reveals the moderating role of perceived inequality in shaping this dynamic. The interaction between personal magnetism and perceived inequality underscores a paradox that is prevalent in societies experiencing high levels of economic disparity: in such contexts, individuals with greater personal magnetism may find that their social appeal exacerbates feelings of inadequacy [50, 51]. This paradox arises from the psychological effects of social comparison processes—where individuals with higher social appeal may engage in upward comparisons that amplify their sense of social and economic deprivation [19, 20]. Thus, perceived inequality does not merely affect material conditions; it disrupts the very social networks that could otherwise provide emotional and instrumental support. This calls for a more nuanced conceptualization of social capital, one that accounts for the socio-economic contexts in which it is embedded and recognizes the ways in which inequality may constrain its psychological benefits.

The role of community trust as a mediating mechanism further complicates the relationship between personal magnetism and depression. The study highlights how personal magnetism, by fostering trust, creates a positive feedback loop that reinforces mental health outcomes. Community trust, in this context, functions not just as a facilitator of social cooperation but also as a psychological resource that mitigates depressive symptoms. Trust-based networks offer individuals emotional and practical support, helping to reduce the sense of social alienation that often accompanies depression [52]. This reinforces the notion that mental health outcomes are shaped by both individual and collective processes: personal magnetism allows individuals to establish trust-based relationships, but it is the broader community context that amplifies or constrains the psychological benefits derived from these networks [53]. As such, community trust acts as a key mediator that strengthens the resilience of individuals, particularly in the face of socio-economic challenges like inequality.

The study’s findings have significant implications for both theoretical and practical understandings of mental health. From a theoretical perspective, they highlight the need to rethink the boundaries between individual agency and social structure in mental health discourse. While personal magnetism undoubtedly contributes to psychological resilience, its effects cannot be fully understood outside the context of the structural conditions that shape its expression. The role of perceived inequality in moderating these effects further emphasizes the importance of considering socio-economic factors in mental health research. This suggests that mental health cannot be fully understood in isolation from the broader social and economic structures in which individuals live, thus calling for a more integrated approach to mental health that incorporates both micro- and macro-level factors.

Third, following Reviewer 2’s suggestions, we have carefully reviewed the manuscript’s structure, making adjustments to ensure logical flow and coherence. Additionally, we have checked the accuracy of the numerical data and refined the presentation of results to enhance clarity. Any identified issues related to grammar, punctuation, and formatting have been corrected.

Thank you again for your constructive feedback.

Best regards.

**Response to Reviewer 1 Comments**

**Point 1:**

Reviewer #1: It was a well-written article that, while focused on China, can be generalized to other developing and developed countries where communities have become increasingly less cohesive. I was unable to find considerable flaws in the data collection, statistics, or interpretations, and agree with conclusions.

**Response 1:**

Dear Reviewer,

We would like to express our sincere gratitude for your encouraging and constructive feedback on our manuscript. We greatly appreciate your positive assessment of our work, particularly regarding the soundness of our data collection, statistical methods, and the validity of our interpretations.

In response to your comments, we have made several refinements to improve the clarity and precision of our arguments. Specifically, we have further developed the Discussion section to emphasize the broader applicability of our findings, particularly in relation to other socio-cultural contexts beyond China. By drawing clearer connections between our study and existing research in both developing and developed countries, we aim to highlight the generalizability of the factors influencing community trust, personal magnetism, and inequality perception.

Moreover, we have addressed the recommendations concerning the logical flow and coherence of the manuscript by refining certain sections, particularly in terms of theoretical alignment and empirical justification. These revisions seek to ensure the internal consistency of the paper while reinforcing the significance of our conclusions in light of the existing literature.

Once again, we deeply appreciate your insightful and thorough review.

Best regards.

**Response to Reviewer 2 Comments**

**Point 1:**

Reviewer #2: The article is well written with a strong scientific approach. The method used (especially statistical approach) is well described and easily comprehensive in a way that allows to reproduce the analysis done. The data as well as the articles mentionned in the ‘References’ support the arguments as well as the conclusion.

Few comments have been made and are available in the attached file.

1. In the abstract and the results section, the number of participants stated is 26711. However, on the 18th page, in the Methods section, it is written 26714 participants.

**Response 1:**

Dear Reviewer,

Thank you very much for your insightful and thorough feedback on our manuscript. We sincerely appreciate your careful attention to detail, particularly in highlighting the discrepancy in the reported sample size between the abstract and the methods section. We are grateful for the opportunity to clarify this issue and provide additional transparency in our methodology.

The initial figure of 26,714 participants, which appears in the abstract section, corresponds to the sample size before the data cleaning process. At that stage, we had not yet conducted the missing data handling procedures, and this figure reflects the total number of observations from the raw dataset across all variables. However, as part of our rigorous data cleaning process, we performed a detailed examination of missing values using the egen miss = rmiss(...) command. This allowed us to identify and address any missing data across key variables, such as depression and personal magnetism.

Following this step, we excluded all observations with any missing values to ensure that our final sample was complete and robust for analysis. This approach is essential for maintaining the integrity of the statistical results, particularly when the missing data may not be randomly distributed, potentially introducing bias. Specifically, the results of the data cleaning process are as follows: miss = 0: No missing values, comprising 26,711 observations (77.57% of the total); miss = 1: Observations with one missing value, comprising only 3 observations (0.01% of the total); miss = 10: Observations with multiple missing values, comprising 7,720 observations (22.42%).

As a result, after excluding all observations with any missing data (whether a single missing value or multiple), the final valid sample size was 26,711. The discrepancy between the reported 26,714 and the final sample size arises from the exclusion of the 3 observations with only a single missing value, which, although minimal, were removed to maintain methodological consistency and ensure the robustness of the dataset.

We understand that this process should have been more clearly articulated in the manuscript, particularly in the Methods section, to ensure full transparency. In response to your comment, we have made the following revisions:

First, we have updated the sample size from 26,714 to 26,711 to ensure consistency across the manuscript, including the abstract, results, and tables.

Second, we have added a brief clarification in the Methods section to explicitly state that 3 observations were excluded due to incomplete data. This clarification ensures full transparency regarding our dataset selection criteria.

**The revised part is as follows:**

**Methods**

**Data and sample**

This study employs data from the 2018 and 2020 waves of the China Family Panel Studies (CFPS), a nationally representative longitudinal survey conducted by the Institute of Social Science Survey at Peking University. The CFPS adopts a stratified multistage probability sampling design, capturing a demographically and geographically diverse sample across 25 provinces, municipalities, and autonomous regions. With data collected from approximately 16,000 households, the CFPS offers a robust empirical basis for analyzing socio-economic and psychological dynamics in contemporary China. The data collection process adhered to rigorous methodological protocols, with trained enumerators conducting computer-assisted personal interviews (CAPI) to enhance data reliability, consistency, and precision.

To ensure the integrity and robustness of the analytical sample, systematic data preprocessing and quality control procedures were applied. Missing data were identified using diagnostic checks and handled through listwise deletion, retaining only cases with complete information across key study variables. The missing data detection process utilized the egen miss = rmiss(...) function, ensuring that excluded cases did not introduce systematic bias. After applying these exclusion criteria, the final analytical sample consisted of 26,711 respondents, ensuring sufficient statistical power for hypothesis testing.

Ethical approval for this study was obtained from the Peking University Ethics Committee, ensuring adherence to the 1964 Helsinki Declaration and subsequent amendments. The study was conducted in accordance with Articles 38, 39, and 40 of the Constitution of the People’s Republic of China, as well as Chapter I, Article 9 of the Statistics Law of the People’s Republic of China, which govern the ethical collection and use of national survey data.

Informed consent was obtained verbally from all respondents prior to data collection. Respondents were provided with a detailed explanation of the study’s objectives, methodology, potential risks, and data confidentiality procedures. To ensure transparency and procedural integrity, enumerators documented the verbal consent process, including time of agreement, reasons for participation or non-participation, and respondent verification. For respondents under the legal age of 18, informed consent was first obtained from their legal guardians, ensuring that all interviews involving minors were conducted under parental supervision in strict compliance with ethical guidelines for research involving vulnerable populations.

Third, we have conducted a comprehensive review of all references to sample size throughout the manuscript, ensuring that 26,711 is consistently reported in all relevant sections.

These revisions address the inconsistency and further clarify our data processing procedures, reinforcing the transparency and reproducibility of our findings.

Once again, we greatly appreciate your thoughtful feedback. Your careful review has strengthened the clarity and reliability of our study, and we are grateful for the opportunity to refine our work based on your insights.

Thank you for your time and valuable contributions to our work.

Best regards.

**Point 2:**

2. Coherence of the article: To improve the flow of the article, I would suggest moving the statistical analysis (below table 1) to a position prior to Table 1, in the Method section.

Additionally, to enhance the coherence of the article, I suggest to introduce Table 1 (descriptive statistics) as the first element in the Results section.

**Response 2:**

Dear Reviewer,

Thank you for your thoughtful and constructive comments on our manuscript. We sincerely appreciate the time and effort you have taken to review our work. Your suggestions for improving the coherence and logical flow of the paper have been extremely valuable, and we have carefully considered them in our revisions.

To enhance the clarity and structure of the manuscript, we have made the following adjustments:

On the one hand, in response to your feedback, we have relocated the statistical analysis section, which was previously placed below Table 1, to the Methods section, directly preceding the presentation of Table 1. This revision ensures that the methodological approach is presented upfront, providing a clearer understanding of the statistical framework used prior to the presentation of descriptive statistics.

**The revised part is as follows:**

**Methods**

**Data and sample**

This study employs data from the 2018 and 2020 waves of the China Family Panel Studies (CFPS), a nationally representative longitudinal survey conducted by the Institute of Social Science Survey at Peking University. The CFPS adopts a stratified multistage probability sampling design, capturing a demographically and geographically diverse sample across 25 provinces, municipalities, and autonomous regions. With data collected from approximately 16,000 households, the CFPS offers a robust empirical basis for analyzing socio-economic and psychological dynamics in contemporary China. The data collection process adhered to rigorous methodological protocols, with trained enumerators conducting computer-assisted personal interviews (CAPI) to enhance data reliability, consistency, and precision.

To ensure the integrity and robustness of the analytical sample, systematic data preprocessing and quality control procedures were applied. Missing data were identified using diagnostic checks and handled through listwise deletion, retaining only cases with complete information across key study variables. The missing data detection process utilized the egen miss = rmiss(...) function, ensuring that excluded cases did not introduce systematic bias. After applying these exclusion criteria, the final analytical sample consisted of 26,711 respondents, ensuring sufficient statistical power for hypothesis testing.

Ethical approval for this study was obtained from the Peking University Ethics Committee, ensuring adherence to the 1964 Helsinki Declaration and subsequent amendments. The study was conducted in accordance with Articles 38, 39, and 40 of the Constitution of the People’s Republic of China, as well as Chapter I, Article 9 of the Statistics Law of the People’s Republic of China, which govern the ethical collection and use of national survey data.

Informed consent was obtained verbally from all respondents prior to data collection. Respondents were provided with a detailed explanation of the study’s objectives, methodology, potential risks, and data confidentiality procedures. To ensure transparency and procedural integrity, enumerators documented the verbal consent process, including time of agreement, reasons for participation or non-participation, and respondent verification. For respondents under the legal age of 18, informed consent was first obtained from their legal guardians, ensuring that all interviews involving minors were conducted under parental supervision in strict compliance with ethical guidelines for research involving vulnerable populations.

**Measures**

**Depression**

Depressive symptoms were assessed using a modified version of the Center for Epidemiologic Studies Depression Scale (CES-D), a widely validated instrument demonstrating high reliability and construct validity in the Chinese population [33,34]. The adapted measure in the CFPS consists of six key items, evaluating the frequency of depressive manifestations, including depressed mood, difficulty initiating activities, sleep disturbances, emotional distress, loneliness, and persistent unhappiness. Each item was measured on a four-point Likert scale, ranging from 1 (rarely or never) to 4 (almost daily). The composite score, derived by summing all responses, represents the severity of depressive symptoms, with higher scores indicative of greater psychological distress. Internal consistency reliability was confirmed, with a Cronbach’s alpha coefficient of 0.76, suggesting acceptable internal reliability for analytical purposes.

**Personal magnetism**

Personal magnetism was operationalized as an individual’s self-perceived social appeal and influence, a construct grounded in social capital and interpersonal attraction theories [35]. The CFPS employed a single-item self-assessment measure, wherein respondents rated their perceived popularity and social influence on a scale from 0 (least magnetic) to 10 (most magnetic). While single-item measures have limitations, prior research suggests that such self-perceptions strongly correlate with broader social capital indicators, making this an efficient proxy for interpersonal influence and embeddedness within social networks [36].

**Inequality perception**

Perceived socioeconomic inequality was measured using a Likert-scale instrument assessing individuals’ subjective evaluation of income disparities in their social environment [37]. Respondents rated their perception of inequality on a scale from 0 (very low inequality) to 10 (very high inequality), reflecting the extent to which they believe income disparities affect their society. This measure aligns with the relative deprivation framework, which posits that perceived inequality—beyond objective economic conditions—can significantly shape social attitudes, psychological well-being, and interpersonal trust [21].

**Community trust**

Community trust was conceptualized as individuals’ perceived reliability and trustworthiness of others within their local community [38]. The CFPS employed a standardized community trust scale, where respondents rated their trust in neighbors and local institutions on a 0 to 10 scale, with higher scores indicating greater confidence in social cohesion and collective efficacy. This measure aligns with social capital theory, which suggests that higher levels of trust are associated with stronger social networks, increased civic engagement, and enhanced psychological well-being [15].

**Control variables**

Control variables included key demographic, socioeconomic, and behavioral factors based on existing literature on social capital and mental health outcomes [39, 40]. These comprised sex (0 = female, 1 = male), age (continuous), marital status (0 = unmarried, 1 = married), education level (1 = below junior high school, 2 = junior high to college, 3 = above college), self-rated health (1 = poor, 5 = excellent), smoking behavior (0 = non-smoker, 1 = smoker), alcohol consumption (0 = non-drinker, 1 = drinker), and household registration (Hukou status, 0 = rural, 1 = urban). The inclusion of these controls accounts for potential biases and ensures robust estimates of the relationships among personal magnetism, community trust, perceived inequality, and depression.

**Statistical analysis**

Data analysis was conducted using Stata version 16 (StataCorp, Texas, USA). A multicollinearity diagnostic test using variance inflation factors (VIFs) was performed to assess potential collinearity among predictors, with all VIF values below 2, indicating no substantial multicollinearity concerns. The main analytical approach consisted of multivariate regression models to examine the direct, moderating, and mediating relationships among key study variables [41-43]. First, ordinary least squares (OLS) regression was employed to estimate the direct association between personal magnetism and depression, controlling for relevant covariates. Second, a moderation analysis was conducted by introducing an interaction term (Personal Magnetism × Perceived Inequality) to determine whether the protective effect of personal magnetism on depression varies with inequality perception levels. Third, a mediation analysis was performed within a structural equation modeling (SEM) framework to assess whether community trust mediates the association between personal magnetism and depression. The Bootstrap method (5,000 resamples) was applied to estimate indirect effects, ensuring statistical robustness and bias correction.

To further examine heterogeneity, subgroup analyses by sex were conducted using stratified regression models to assess whether the observed relationships differed between male and female respondents. Additionally, several robustness checks were performed, including alternative model specifications using a fixed-effects ordered logit model and the inclusion of additional control variables (e.g., trust in strangers). These robustness tests confirmed the consistency of the findings across different analytical approaches, reinforcing the validity of the proposed theoretical framework. This comprehensive methodological approach ensures rigorous hypothesis testing and enhances the generalizability of the study’s conclusions regarding the interplay between personal magnetism, community trust, perceived inequality, and depression in contemporary China.

On the other hand, following your suggestion, we have repositioned Table 1 (descriptive statistics) to the beginning of the Results section. This structural change ensures that the data are introduced first, setting a solid foundation for the ensuing analysis. By presenting the descriptive statistics upfront, we aim to provide readers with a clear and coherent starting point that will guide them through the more complex findings that follow.

**The revised part is as follows:**

**Results**

**Descriptive analysis**

Table 1 presents the descriptive statistics for the key study variables. The mean depression score across the sample is 13.43 (S. D. = 3.72, range: 8–32), indicating moderate levels of depressive symptoms. Personal magnetism has a mean of 6.99 (S. D. = 1.80, range: 0–10), suggesting that respondents, on average, perceive themselves as moderately socially influential. Inequality perception exhibits a mean of 7.19 (S. D. = 2.27, range: 0–10), highlighting a relatively high awareness of economic disparity within the population. Community trust averages 6.56 (S. D. = 2.04, range: 0–10), reflecting moderate levels of perceived trust in one’s community, though with notable individual variation.

**Table 1.** Descriptive statistics of all variables, CFPS 2018-2020 (N = 26,711).

| **Variable** | **Mean (SD)** | **Range** | **Low** | **Moderate** | **High** | **Category** | **% (N)** |
| --- | --- | --- | --- | --- | --- | --- | --- |
| Depression | 13.43 (3.72) | 8 - 32 | 8 - 12 | 13 - 19 | 20 - 32 |  |  |
| Personal Magnetism | 6.99 (1.80) | 0 - 10 | 0 - 3 | 4 - 7 | 8 - 10 |  |  |
| Inequality Perception | 7.19 (2.27) | 0 - 10 | 0 - 3 | 4 - 7 | 8 - 10 |  |  |
| Community Trust | 6.56 (2.04) | 0 - 10 | 0 - 3 | 4 - 7 | 8 - 10 |  |  |
| Age | 33.53 (11.89) | 16 - 96 | 16 - 22 | 23 - 44 | 45 - 96 |  |  |
| Self-Rated Health | 3.33 (1.10) | 1 - 5 | 1 - 2 | 3 | 4 - 5 |  |  |
| Sex |  | 0 - 1 |  |  |  | Female | 50.20% (13,409) |
|  |  |  |  |  |  | Male | 49.80% (13,302) |
| Marital Status |  | 0 - 1 |  |  |  | Unmarried | 32.02% (8,553) |
|  |  |  |  |  |  | Married | 67.98% (18,158) |
| Education Level |  | 1 - 3 |  |  |  | Below Junior High | 23.37% (6,242) |
|  |  |  |  |  |  | Junior High to College | 65.47% (17,487) |
|  |  |  |  |  |  | Above College | 11.16% (2,982) |
| Smoking |  | 0 - 1 |  |  |  | Non-smoker | 72.93% (19,480) |
|  |  |  |  |  |  | Smoker | 27.07% (7,231) |
| Alcohol Drinking |  | 0 - 1 |  |  |  | Non-drinker | 88.65% (23,678) |
|  |  |  |  |  |  | Drinker | 11.35% (3,033) |
| Residence Registration |  | 0 - 1 |  |  |  | Rural | 74.71% (19,957) |
|  |  |  |  |  |  | Urban | 25.29% (6,754) |

Regarding demographic variables, the mean age of respondents is 33.53 years (SD = 11.89, range: 16–96), with the majority falling within the 23–44 age range. A near-equal distribution of sex is observed, with 50.20% of respondents being female and 49.80% male. Marital status distribution indicates that 67.98% of respondents are married, whereas 32.02% remain unmarried. Educational attainment is stratified into three levels, with 23.37% of respondents having an education below junior high school, 65.47% possessing an education level between junior high and college, and 11.16% holding a degree beyond college level.

Health and lifestyle variables provide further context for the sample composition. Self-rated health averages 3.33 (S. D. = 1.10, range: 1–5), with most individuals rating their health as moderate. Smoking behavior is reported by 27.07% of respondents, while 11.35% consume alcohol regularly. A significant rural-urban divide is evident, with 74.71% of respondents residing in rural areas and 25.29% in urban locations. These distributions offer foundational insights into the socio-demographic structure of the sample and its implications for the study’s core variables.

These revisions have been made with the intent of improving the overall flow of the manuscript, ensuring that the structure supports a logical and seamless progression from the methods section to the results.

Once again, we are grateful for your insightful suggestions, which have contributed to the refinement of our manuscript.

Best regards.

**Point 3:**

3. Easier comprehension of the descriptive statistics : Include a table or additional text that provides a more detailed breakdown of the ranges for the various key study variables, such as depression, personal magnetism, community trust, and perceived social allure. Specifically, the table or text could outline the score ranges (from 0 to a specified upper limit) for each variable and categorize them into distinct levels (e.g., low, moderate, high). This would offer readers a clearer understanding of how the variables are quantified and help contextualize the results more effectively.

**Response 3:**

Dear Reviewer,

We sincerely appreciate your insightful suggestion regarding the need for a more comprehensive breakdown of the descriptive statistics in the manuscript. Your feedback has significantly contributed to improving the clarity and interpretability of our study, especially in terms of how key variables such as depression, personal magnetism, community trust, and inequality perception are contextualized and understood.

In response to your valuable comments, we have made the following revisions to enhance the transparency and explanatory power of Table 1:

First, we have revised Table 1 to provide a more detailed and explicit breakdown of the descriptive statistics for each key variable. Specifically, we now include the range of scores for each variable, providing the minimum and maximum values to offer transparency in how each variable was measured. Additionally, we have categorized each variable into low, moderate, and high levels, based on both theoretical and empirical considerations.

Second, The classification of each key variable into low, moderate, and high levels was determined by the empirical distribution of responses within the sample (N = 26,711). This ensures that each category accurately reflects meaningful groupings while maintaining statistical robustness.

For depression, the scores ranged from 8 to 32, with a mean of 13.43 (SD = 3.72), and a majority of responses clustered between 13 and 19, forming a nearly normal distribution. Based on this, we classified: mild depression (8-12) as below 1 SD from the mean, moderate depression (13-19) as the central 68% of observations, severe depression (20-32) as exceeding 1 SD above the mean.

For personal magnetism, the scores (Mean = 6.99, SD = 1.80) were concentrated between 4 and 7, with fewer respondents at the extremes (0-3 and 8-10). Consequently, we classified: low personal magnetism (0-3), moderate (4-7), high (8-10).

For inequality perception, with a mean of 7.19 (SD = 2.27), the distribution was right-skewed, with most responses falling between 4 and 9. As such, we classified: low inequality perception (0-3), capturing the bottom 15-20% of responses, moderate inequality perception (4-7), covering the central 60-70%, high inequality perception (8-10), representing the upper tail.

For community trust, the mean was 6.56 (SD = 2.04), and the distribution showed a slight negative skew, with most responses falling between 4 and 9. Therefore, we classified: low community trust (0-3), moderate trust (4-7), high trust (8-10).

These classifications were grounded in the natural empirical distribution of the data, which ensures both statistical validity and interpretability, while avoiding the imposition of arbitrary cutoffs.

Third, we have also added an accompanying narrative in the manuscript that discusses the distributional characteristics of these variables, providing a deeper contextual interpretation. This addition highlights how variations in depression, personal magnetism, inequality perception, and community trust can influence broader social behaviors, well-being, and perceptions of inequality. This interpretation strengthens the relevance of the data and its implications for understanding social dynamics within the studied community.

**The revised part is as follows:**

**Results**

**Descriptive analysis**

Table 1 presents the descriptive statistics for the key study variables. The mean depression score across the sample is 13.43 (S. D. = 3.72, range: 8–32), indicating moderate levels of depressive symptoms. Personal magnetism has a mean of 6.99 (S. D. = 1.80, range: 0–10), suggesting that respondents, on average, perceive themselves as moderately socially influential. Inequality perception exhibits a mean of 7.19 (S. D. = 2.27, range: 0–10), highlighting a relatively high awareness of economic disparity within the population. Community trust averages 6.56 (S. D. = 2.04, range: 0–10), reflecting moderate levels of perceived trust in one’s community, though with notable individual variation.

**Table 1.** Descriptive statistics of all variables, CFPS 2018-2020 (N = 26,711).

| **Variable** | **Mean (SD)** | **Range** | **Low** | **Moderate** | **High** | **Category** | **% (N)** |
| --- | --- | --- | --- | --- | --- | --- | --- |
| Depression | 13.43 (3.72) | 8 - 32 | 8 - 12 | 13 - 19 | 20 - 32 |  |  |
| Personal Magnetism | 6.99 (1.80) | 0 - 10 | 0 - 3 | 4 - 7 | 8 - 10 |  |  |
| Inequality Perception | 7.19 (2.27) | 0 - 10 | 0 - 3 | 4 - 7 | 8 - 10 |  |  |
| Community Trust | 6.56 (2.04) | 0 - 10 | 0 - 3 | 4 - 7 | 8 - 10 |  |  |
| Age | 33.53 (11.89) | 16 - 96 | 16 - 22 | 23 - 44 | 45 - 96 |  |  |
| Self-Rated Health | 3.33 (1.10) | 1 - 5 | 1 - 2 | 3 | 4 - 5 |  |  |
| Sex |  | 0 - 1 |  |  |  | Female | 50.20% (13,409) |
|  |  |  |  |  |  | Male | 49.80% (13,302) |
| Marital Status |  | 0 - 1 |  |  |  | Unmarried | 32.02% (8,553) |
|  |  |  |  |  |  | Married | 67.98% (18,158) |
| Education Level |  | 1 - 3 |  |  |  | Below Junior High | 23.37% (6,242) |
|  |  |  |  |  |  | Junior High to College | 65.47% (17,487) |
|  |  |  |  |  |  | Above College | 11.16% (2,982) |
| Smoking |  | 0 - 1 |  |  |  | Non-smoker | 72.93% (19,480) |
|  |  |  |  |  |  | Smoker | 27.07% (7,231) |
| Alcohol Drinking |  | 0 - 1 |  |  |  | Non-drinker | 88.65% (23,678) |
|  |  |  |  |  |  | Drinker | 11.35% (3,033) |
| Residence Registration |  | 0 - 1 |  |  |  | Rural | 74.71% (19,957) |
|  |  |  |  |  |  | Urban | 25.29% (6,754) |

Regarding demographic variables, the mean age of respondents is 33.53 years (SD = 11.89, range: 16–96), with the majority falling within the 23–44 age range. A near-equal distribution of sex is observed, with 50.20% of respondents being female and 49.80% male. Marital status distribution indicates that 67.98% of respondents are married, whereas 32.02% remain unmarried. Educational attainment is stratified into three levels, with 23.37% of respondents having an education below junior high school, 65.47% possessing an education level between junior high and college, and 11.16% holding a degree beyond college level.

Health and lifestyle variables provide further context for the sample composition. Self-rated health averages 3.33 (S. D. = 1.10, range: 1–5), with most individuals rating their health as moderate. Smoking behavior is reported by 27.07% of respondents, while 11.35% consume alcohol regularly. A significant rural-urban divide is evident, with 74.71% of respondents residing in rural areas and 25.29% in urban locations. These distributions offer foundational insights into the socio-demographic structure of the sample and its implications for the study’s core variables.

We truly appreciate your thoughtful and constructive feedback on improving the coherence and accessibility of our results. Thank you again for your careful review and for contributing to the refinement of our work.

Best regards.

**Point 4:**

4. Define the age range of the adults included in the study.

**Response 4:**

Dear Reviewer,

We deeply appreciate your constructive feedback regarding the need to clarify the age range in our study. Your comment has provided an opportunity to further enhance the transparency and robustness of our methodology, and we have made the necessary revisions to address your concerns.

The full sample in our study includes individuals aged 16 to 96 years (Mean = 33.53, SD = 11.89, N = 26,711), representing a wide demographic range. This broad age spectrum was chosen to capture a comprehensive view of the population, but we recognize the importance of verifying the robustness of our findings, particularly in relation to specific age cohorts that are more directly relevant to the working-age population.

To ensure the validity of our conclusions and to account for any potential age-related biases, we conducted a supplementary analysis by restricting the sample to individuals aged 16 to 59. This age segmentation is grounded in practical considerations and empirical patterns specific to China’s socio-economic landscape. In the Chinese context, 16 is a key threshold, as individuals are legally allowed to enter the labor market, often through vocational training or apprenticeships, and begin their economic participation. Similarly, 59 represents a pre-retirement age, in line with the country’s labor policies, where most individuals—especially men—are expected to retire by the age of 60, with many women retiring even earlier.

The rationale behind excluding individuals above the age of 59 is based on the demographic and labor force dynamics, where individuals over 60 typically exit the formal workforce and transition to roles in family-based caregiving or informal economic participation, which differ substantially from the patterns associated with active employment. By focusing on this core working-age group, we ensured that our key findings were not unduly influenced by the inclusion of older individuals, and the observed patterns remained statistically stable and socially meaningful within this restricted age range.

Furthermore, we acknowledge the presence of respondents under the legal age of 18 in our dataset. To address any potential ethical concerns, we explicitly state that all survey responses from minors were obtained with explicit parental or guardian consent, in full compliance with China’s ethical and legal standards for research involving minors. The CFPS survey administrators took the necessary steps to ensure that minors fully understood the nature of the study before participation, and their guardians provided informed consent.

In response to your suggestion, we have now clarified these age-related considerations in the revised manuscript to enhance the methodological transparency and the empirical validity of our findings.

**The revised part is as follows:**

**Robustness test**

To validate the robustness of the findings, several adjustments were made to the sample and model specifications. First, the analysis was re-conducted excluding individuals aged 60 and above, as mental health assessments may differ for this age group due to factors such as physiological changes, retirement, and social role transitions [44, 45]. The original sample included individuals aged 16 to 96 years. The results derived from the modified sample, now limited to individuals aged 16 to 59, were consistent with those from the full sample, thereby reinforcing the robustness of the initial findings.

Second, the fixed-effects regression model was replaced with an ordered logit (ologit) model to account for the ordinal nature of the depression outcome variable [46]. Specifically, the outcome variable was constructed to categorize depression scores into three distinct levels: low (8–12), moderate (13–19), and high (20–32). This categorical classification allowed for a more nuanced analysis of the depression outcomes. This substitution did not alter the substantive conclusions, reinforcing the robustness of the findings across model specifications.

Third, an additional control variable, trust in strangers, was introduced to examine its potential influence on depression, as interpersonal trust is a crucial determinant of social interactions and mental health outcomes [47]. This variable was measured on a scale ranging from 0 (very low trust) to 10 (very high trust). The incorporation of trust in strangers into the model did not significantly alter the results, further substantiating the robustness of the primary findings. Taken together, these robustness checks confirm the stability of the results across different specifications and sample modifications, thereby enhancing the credibility and generalizability of the conclusions.

We sincerely appreciate your valuable suggestion, which has helped us refine the clarity and contextual relevance of our research in meaningful ways.

Best regards.

**Point 5:**

5. For additional comments in the attached file:

*** The time of agreement / agreeing *** Disagreement

**Response 5:**

Dear Reviewer,

We sincerely appreciate your careful review and the detailed feedback regarding the wording in the informed consent section of our manuscript. Your suggestions have been invaluable in refining the clarity and precision of our language. We have carefully considered your feedback and made the necessary revisions to enhance the accuracy of our phrasing.

In response to your suggestions, we have made the following adjustments:

First, we have revised this phrase to “the time at which consent was given,” ensuring both grammatical correctness and greater clarity in the description of the informed consent process.

Second, We have replaced the term “disagreement” with “the reasons for refusal,” as this more accurately reflects the nature of participants’ decision to not engage in the study. This modification aims to convey the intended meaning with greater precision and transparency.

Furthermore, we have explicitly clarified the informed consent process for minors. Specifically, we have stated that consent was first obtained from the guardian, and minors participated in the study under their guardians' supervision, fully adhering to ethical and legal standards for research involving individuals under the age of 18.

**The revised part is as follows:**

**Informed consent**

Verbal informed consent was obtained from all individual respondents included in the study. The design of this survey complied with Articles 38, 39, and 40 of the Constitution of the People’s Republic of China and was conducted within the legal framework outlined in Chapter I, Article 9 of the Statistics Law of the People’s Republic of China. Verbal informed consent was deemed acceptable and was not subject to review by an ethics committee.

Additionally, interviewers documented detailed information regarding the informed consent process, including whether the respondent agreed to participate in the study, the time at which consent was given, and, in cases of non-participation, the reasons for refusal. Furthermore, for respondents under the legal age of 18, informed consent was first obtained from their legal guardians, who were fully informed about the study’s purpose and procedures. The participation of minors was conducted under the supervision of their guardians, ensuring compliance with ethical and legal standards for research involving minors. All records related to informed consent were securely stored by Peking University.

These revisions enhance the accuracy and transparency of our informed consent description while ensuring alignment with international research ethics standards. We appreciate your valuable input, which has contributed to improving the clarity of our manuscript.

Best regards.
